# High-throughput *Saccharomyces cerevisiae* cultivation method for credentialing-based untargeted metabolomics

**DOI:** 10.1007/s00216-023-04724-5

**Published:** 2023-05-22

**Authors:** Lorenzo Favilli, Corey M. Griffith, Emma L. Schymanski, Carole L. Linster

**Affiliations:** Luxembourg Centre for Systems Biomedicine (LCSB), University of Luxembourg, Avenue du Swing 6, Belvaux, L-4367 Luxembourg

**Keywords:** High-throughput sample generation, Liquid chromatography, Metabolomics, *Saccharomyces cerevisiae*, Stable isotope labelling, Untargeted high-resolution mass spectrometry

## Abstract

Identifying metabolites in model organisms is critical for many areas of biology, including unravelling disease aetiology or elucidating functions of putative enzymes. Even now, hundreds of predicted metabolic genes in *Saccharomyces cerevisiae* remain uncharacterized, indicating that our understanding of metabolism is far from complete even in well-characterized organisms. While untargeted high-resolution mass spectrometry (HRMS) enables the detection of thousands of features per analysis, many of these have a non-biological origin. Stable isotope labelling (SIL) approaches can serve as credentialing strategies to distinguish biologically relevant features from background signals, but implementing these experiments at large scale remains challenging. Here, we developed a SIL-based approach for high-throughput untargeted metabolomics in *S. cerevisiae*, including deep-48 well format-based cultivation and metabolite extraction, building on the peak annotation and verification engine (PAVE) tool. Aqueous and nonpolar extracts were analysed using HILIC and RP liquid chromatography, respectively, coupled to Orbitrap Q Exactive HF mass spectrometry. Of the approximately 37,000 total detected features, only 3–7% of the features were credentialed and used for data analysis with open-source software such as MS-DIAL, MetFrag, Shinyscreen, SIRIUS CSI:FingerID, and MetaboAnalyst, leading to the successful annotation of 198 metabolites using MS^2^ database matching. Comparable metabolic profiles were observed for wild-type and *sdh1*Δ yeast strains grown in deep-48 well plates versus the classical shake flask format, including the expected increase in intracellular succinate concentration in the *sdh1*Δ strain. The described approach enables high-throughput yeast cultivation and credentialing-based untargeted metabolomics, providing a means to efficiently perform molecular phenotypic screens and help complete metabolic networks.

**Graphical abstract:**

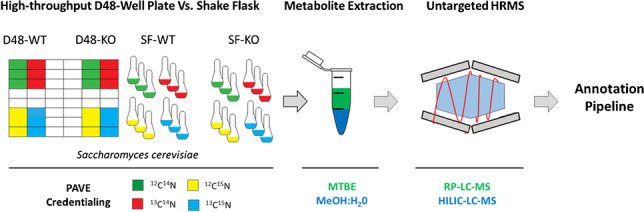

**Supplementary Information:**

The online version contains supplementary material available at 10.1007/s00216-023-04724-5.

## Introduction

*Saccharomyces cerevisiae* (*S. cerevisiae*, budding yeast) is a unicellular, eukaryotic model organism that is well-suited for discovering key cellular processes and even studying mechanisms of human disease due to its genetic malleability, the availability of genome wide knockout (KO) collections, and the considerable conservation between yeast and human genes [1, 2]. Compellingly, thousands of sequenced genes remain uncharacterized in model organisms. In 2017, it was estimated that the biological function of 29% of the *S. cerevisiae* proteome was still unknown, with a significant proportion suspected to have catalytic functions [2]. Metabolomics, a technique enabling the comprehensive study of metabolic networks and metabolic states of organisms, is routinely performed using gas chromatography (GC) or liquid chromatography (LC) coupled to mass spectrometry [3]. Advancements in high-resolution mass spectrometry (HRMS) have expanded our understanding of biochemical metabolic networks, yet the large number of remaining enzymes of unknown function indicates that there are likely still many hidden or unknown metabolic reactions and hence metabolites. HRMS instrumentation offers high mass resolving power and mass accuracy, which is ideal for identifying novel metabolites with high confidence [4]. HRMS data often are acquired using data-dependent acquisition (DDA), where a selected number of ions are fragmented using tandem mass spectrometry (MS^2^) at a given time to obtain a chemical fingerprint of the ion, which can be pieced together like a puzzle to reveal its structure. Large community efforts have established open source MS^2^ libraries (e.g. MassBank) [5] and data processing software (e.g. MS-DIAL [6], XCMS [7], and Open MS [8]) [9, 10] to assist in metabolite annotation. Cheminformatics and in silico fragmentation tools are commonly used to overcome the gap between available experimental MS^2^ and the reported chemical space by retrieving candidates from compound databases and ranking them using in silico methods [10, 11]. Widely used in silico fragmentation prediction approaches include MetFrag [12], Sirius CSi:FingerID [13, 14], and LipidBlast [15]. These, combined with compound databases such as HMDB [16, 17], YMDB [18, 19], KEGG [20], and PubChemLite [11], assist feature annotation and help to condense, filter, and organize the obtained results [9–11]. Untargeted HRMS-based metabolomics is a valuable approach for elucidating the biochemical roles of unknown enzymes and completing metabolic networks. In particular, the ex vivo metabolic profiling approach [2, 21] has been used for functional investigations of unknown enzymes based on the analysis of overexpression and/or knockout strains of the gene of interest, including notable examples in budding yeast [22–26].

The intracellular formation of non-canonical metabolites adds another layer of complexity to metabolome annotation efforts. In contrast to the historical viewpoint that metabolic enzymes are highly specific, it is now clear that non-canonical metabolites arise from enzyme promiscuity and non-enzymatic reactions, thereby increasing the chemical diversity of the metabolic space. Under normal conditions, the concentration of these non-canonical metabolites is usually maintained at very low levels by dedicated metabolite repair enzymes, which reconvert the useless or potentially toxic metabolic side products to useful and/or benign products [27, 28]. Deficiencies in metabolite repair enzymes can lead to inherited metabolic disorders, and enzyme promiscuity and metabolite repair possibilities are important considerations in metabolic engineering endeavours [29–32]. It is anticipated that metabolite repair enzymes could make up a considerable portion of the remaining enzymes of unknown function, since there should be relatively few gaps remaining in primary metabolic pathways [2]; untargeted metabolomics approaches are a valuable asset to also elucidate this (often neglected) part of metabolism.

However, of the tens of thousands of features classically detected by untargeted HRMS metabolomics analysis in biological samples, over 90% are likely not of (direct) biological origin but rather in-source fragments, adducts, isotopes, environmental contaminants, and other artefacts [33]. In addition, approximately only 2% of the detected features are commonly annotated, leaving the vast majority of the collected information uncharacterized (the so-called dark matter [34]). Often, it is not possible to distinguish between background and truly biological signals in conventional untargeted workflows, leading to important peaks being overlooked, along with annotation (or misannotation) of less relevant background features and potentially erroneous biological interpretations. Credentialing strategies [35] are designed to unearth biologically derived features from background by comparing data obtained from unlabelled and stable isotope-labelled metabolite extracts. Here, microbial cultures can be grown in identical conditions using unlabelled or stable isotope-labelled substrates (e.g. glucose-^13^C_6_, (^15^NH_4_)_2_SO_4_), and the metabolite extracts are analysed by GC-MS or LC-MS [28]. Various software approaches (e.g. IROA [36], X^13^CMS [37], mzMatch-ISO [38], geoRge [39], MetExtractII [40]) and the Peak Annotation and Verification Engine (PAVE) [41] are available to identify mass shifts (corresponding to the number of labelled atoms) at a given retention time (RT). Credentialing reduces the tens of thousands of features typically detected in an untargeted experiment to hundreds or thousands of biologically relevant ones [35, 41], which can then be prioritized for annotation and biological interpretation. The latter still represent major bottlenecks of metabolomics studies together with metabolite coverage and analytical throughput [42]. Credentialing is particularly appealing to perform with prototrophic microorganisms where uniformly labelled extracts can be obtained, as highlighted in the PAVE workflow [41]. PAVE compares metabolite extracts of cells cultivated in unlabelled, ^13^C, ^15^N, and ^13^C+^15^N media and injected separately to identify and remove adducts, isotopes, MS artefacts, and in-source fragments. The resulting peak list contains only biologically derived features (i.e. those features where the stable isotopes have been integrated), which are assigned carbon/nitrogen counts and, in some cases, molecular formulas. When applied to microorganisms, PAVE successfully credentialed between 2 and 5% of the features detected in *Escherichia coli* and *S. cerevisiae* extracts, while the rest of the detected signals were recognized as non-biological with the majority arising from background signals (80%), along with adducts (4%), and isotopes (4%). Over 200 credentialed features were subsequently annotated using internal standards and mass-to-charge ratio (*m/z*), retention time (RT), and MS^2^ spectral matches (148 by RT and *m/z*, 73 with additional MS^2^ match).

Credentialing dramatically increases the experimental complexity, sample number, analytical time, and cost of metabolomics experiments. High-throughput, multi-well cultivation methods may alleviate the experimental effort and enable testing of multiple strains and/or conditions in a single experiment [43–47], ultimately making large-scale credentialing experiments feasible. For instance, Ewald and colleagues (2009) [44] used a multi-well format for cultivation, quenching, and quantification of 30 primary yeast metabolites using GC-TOF. Using a vacuum manifold, fast quenching of metabolism in the exponential growth phase was achieved by transferring the cultivation broth of a 96-well fritted plate into a 48-well plate containing pre-cooled methanol (-40 °C). The validity of the method was supported by the highly comparable results observed in multi-well and shake flask format in terms of growth rate, substrate uptake, by-product formation, and metabolic profiles.

Although high-throughput cultivation is a promising approach for performing large-scale metabolomics, relatively few studies of this type are reported in the literature and to the best of our knowledge, none of the reported studies have integrated a high-throughput labelling strategy with untargeted metabolic profiling. Herein, a high-throughput *S. cerevisiae* cultivation method in a deep-48 well (D48) format is presented that enables credentialing-based untargeted metabolomics using hydrophilic interaction liquid chromatography (HILIC)-HRMS and lipid analyses using reverse phase (RP) LC-HRMS. Yeast strains were simultaneously cultivated in unlabelled or uniformly labelled (^13^C, ^15^N, and ^13^C+^15^N) conditions, and the presented robust, easy-to-handle, and efficient experimental workflow allowed for screening of multiple conditions and/or strains and generation of 48 polar and nonpolar extracts for LC-HRMS analysis per experiment. A computational workflow based on MS-DIAL and MetFrag combined with PubChemLite, Sirius CSI:FingerID, and MetaboAnalyst [48] was established. The openly accessible R package Shinyscreen [49, 50] was used to perform automated mass shift quality control for credentialed results, including pre-screening of MS data with quality control of MS^1^ and MS^2^ event alignment and automated MS^2^ spectra extraction. Analogous to Ewald and co-authors [44], we compared metabolic profiles between a strain with a metabolic enzyme gene deletion (*sdh1*Δ) and a wild-type control strain as a case study and expanded their proof-of-principle for biological application using a hypothesis-generating untargeted approach. The highly comparable results obtained with the D48 well format and classical shake flask (SF) approaches, at both the cultivation and analytical levels, support that the proposed workflow for high-throughput credentialing-based untargeted metabolomics in yeast will push the outcome and quality of metabolic phenotypic screening efforts in this model organism to the next level.

## Materials and methods

### Experimental pipeline

#### Yeast cultivation

The prototrophic *S. cerevisiae* strains (*MATa can1∆::STE2pr-SpHIS5 his3∆1 lyp1∆0 ho*^*−*^) were kindly provided by Prof. Joseph Schacherer [51]. The KO strain (*sdh1*Δ) used had the *SDH1* gene (encoding the flavoprotein subunit of succinate dehydrogenase) replaced by the kanamycin resistance cassette (kanMX). A strain with the kanMX cassette in the *HO* gene was used as the wild-type (WT) control strain. Yeast strains were cultivated in filter-sterilized minimal yeast nitrogen base (5 g/L) medium without ammonium sulphate (YNB w/o ammonium sulphate, MP Biochemicals) containing 20 g/L D-glucose (Sigma) and 1.7 g/L ammonium sulphate (Sigma), and the pH was adjusted to 5.5 (this medium is hereafter designated as ^12^C-YNB medium). D-Glucose was replaced with uniformly carbon labelled D-glucose (20 g/L, U-^13^C_6_, 99%, Cambridge Isotope Laboratories Inc.) in the ^13^C-YNB and ^13^C^15^N-YNB conditions, while the ammonium sulphate was replaced with uniformly nitrogen labelled ammonium sulphate (1.7 g/L, ^15^N_2_SO_4_, 99%, Cambridge Isotope Laboratories Inc.) in ^15^N-YNB and ^13^C^15^N-YNB conditions.

Yeast glycerol stock solutions [23] were used to inoculate ^12^C-YNB cultures with a single colony of the respective strains from agar plates (20 g/L agar, 20 g/L D-glucose, 6.7 g/L YNB with ammonium sulphate) after incubation of minimum 3 days at 30 °C. For a complete experiment, 12 single colonies of each strain were used to inoculate 5-mL pre-cultures for the four media conditions (^12^C-YNB, ^13^C-YNB, ^15^N-YNB, and ^13^C^15^N-YNB) in 14-mL cell culture tubes (CELLSTAR® Cell Culture Tubes, Greiner bio-one) that were shaken at 30 °C and 200 rpm (Infors HT Multitron Standard). The cell densities of the pre-cultures were measured 24 h after inoculation to set the starting OD_600_ of the main cultures (in D48 plates or SF) to 0.025. For the D48 plates (Axygen, 5 mL 48 rectangular wells, V-bottom, P-5ML-48-C-D), 4-mm glass beads were added to each well in order to improve the mixing [44]. For the presented work, twenty-four wells of the D48 plates were filled with 3 mL of each main culture. The remaining wells were filled with either sterile YNB medium (without carbon or nitrogen source, *n* = 8) or sterile ^12^C-YNB (*n* = 16) to prepare extraction blanks (the glucose-free YNB medium blanks were used for the PAVE data analysis; ^12^C-YNB blanks were used to estimate cross-over between wells during cultivation and metabolite extraction). The D48 plates were sealed with a gas-permeable lid (AeraSeal film, Sigma-Aldrich), and the cultivation was conducted at 30 °C and shaken at 400 rpm (Edmund Bühler, TiMix 2). The SF cultivations were performed in 250 mL Erlenmeyer flasks filled with 25 mL medium at 30 °C and shaken at 200 rpm (Infors HT Multitron Standard).

Using the above-described growth conditions, and in an independent experiment, cell concentrations and extracellular glucose levels were measured hourly to estimate the growth and glucose uptake rate of the yeast WT strain for the SF and D48 cultivation method in the ^12^C-YNB medium. Cell concentration was measured using a Multisizer Z3 Coulter Counter (30 μm measurement capillary, Beckman Coulter) after dilution in ISOTON II solution (Beckman Coulter). The substrate consumption was measured in sterile-filtered (0.2 μm cellulose syringe filter, VWR Chemicals) spent media that were stored at  − 20 °C until D-glucose measurement using a YSI (Yellow Springs Instruments 2900 Series Biochemistry Analyser).

#### Sampling and extraction of intracellular metabolites

Cell pellets were harvested during the exponential growth phase (16 h) using a fast centrifugation treatment [52]. For the D48 approach, prior to centrifugation, 200 μL of culture per well was transferred, using a multichannel pipette (E4 XLS, 8 channel electronic pipette, 100–1200 *μ*L*,* Rainin), to another D48 plate containing 2.8 mL ISOTON II solution per well for the biovolume measurements (also referred to as *Measured* *Biovolume* [*μ*L/mL]) using the Multisizer Z3 Coulter Counter. The D48 plates were centrifuged for 20 s at 4 °C and 4816 g (Heraeus Multifuge × 3R, Thermo Scientific), the supernatant was discarded by plate inversion, and the cell pellets were flash-frozen by placing the plates in liquid nitrogen. The above-described sampling procedure using the D48 approach took approximately 6 min, per D48 plate, i.e. 7.5 s per sample. Analogously, 1 mL of the SF cultivations was sampled for biovolume measurements, and 2-mL aliquots was transferred to fresh 2-mL Eppendorf tubes and centrifuged for 20 s at 4 °C and 16000 g (Centrifuge 5415 R, Eppendorf). The supernatants were discarded, and cell pellets were flash-frozen in liquid nitrogen. The sampling time for the SF cultivation method was approximately 16 min for 27 samples (24 biological samples representing three biological replicates for each of the WT and KO strains in the four cultivation conditions needed in the PAVE approach and three YNB glucose-free extraction blanks) or 35.5 s per sample. For both experimental setups, metabolites were extracted using biphasic liquid–liquid extraction (MTBE:MeOH:H_2_O 65:20:15) [53]. For the D48 plates, 635 μL of MeOH:H_2_O mixture (55:45,  − 20 °C) was added to the pellets. Cells were resuspended by shaking at 1000 rpm for 5 min at room temperature (Thermomixer Comfort, Eppendorf) and 1154 μL MTBE (− 20 °C) was added for metabolite extraction. The plates were covered with an empty D48 plate, sealed with parafilm (PARAFILM® M, Merck) and tape, and incubated for 2 h at 4 °C and shaken at 700 rpm (Thermomixer Comfort). The whole cell lysates were transferred to 2-mL Eppendorf tubes for subsequent phase separation. During this extraction procedure, all pipetting steps were performed using a multichannel pipette for the D48 approach. The same metabolite extraction procedure and extraction fluid volumes were applied to the SF samples but through manual pipetting. The extraction procedure for the D48 approach took approximately 12 min per D48 plate (i.e. 15 s per sample), compared to approximately 18 min for 27 samples in the SF approach (i.e. 40 s per sample). For both methods, phase separation between the upper, nonpolar phase (MTBE), and the lower aqueous phase (MeOH:H_2_O) was achieved by centrifugation for 10 min at 4 °C and 16000 g (Centrifuge 5415 R, Eppendorf). Subsequently, 750 μL and 300 μL of the upper nonpolar and lower aqueous phase, respectively, were transferred to 1.5-mL Eppendorf tubes. To improve evaporation, 200 μL MeOH were added to the nonpolar phase, and all metabolite extracts were dried overnight in a SpeedVac at  − 4 °C (Labconco).

#### LC-HRMS analyses

Adapted from already published work [54, 55], the dried polar extracts were resuspended in 80:20 ACN:H_2_O containing 10 μM 4-chloro-L-phenylalanine (Sigma-Aldrich) as the internal standard, while the dried nonpolar extracts were reconstituted in 90:10 MeOH:toluene containing 440 nM 12-[(cyclohexylcarbamoyl)amino]dodecanoic acid (Sanbio) as the internal standard. Samples were normalized by adjusting the *Resuspension Volume* [mL] to obtain a *Fixed Biovolume* of 10 μL/mL using the *Measured Biovolume* [μL/mL] as follows:

$$Resuspension\;Volume\;\lbrack\mathrm{mL}\rbrack=\frac{Measured\;Biovolume\;\left[\frac{\mu L}{\mathrm{mL}}\right]\times Sampling\;Volume\;\left[\mathrm{mL}\right]\times Collected\;Phase\;\lbrack\mathrm{mL}\rbrack}{Extraction\;Fluid\;Volume\;\left[\mathrm{mL}\right]\times Fixed\;Biovolume\;\lbrack\frac{\mu\mathrm L}{\mathrm{mL}}\rbrack}$$

The *Sampling Volume* [mL] refers to the collected culture aliquots (e.g. 2.8 mL from the D48 plates and 2 mL from the SFs), the *Collected Phase* [mL] is 0.75 mL for the organic and 0.3 mL for the aqueous phase, and the *Extraction Fluid Volume* [mL] refers to the added organic solvent (1.154 mL MTBE for the organic/nonpolar phase, 0.635 mL MeOH:H_2_O for the aqueous phase).

The obtained cell extracts, after resuspension to 10 μL/mL biovolume, were diluted further to a final biovolume of 7.5 μL/mL for injection. Samples were centrifuged for 10 min at 4 °C and 16000 g (Centrifuge 5415 R, Eppendorf), and 50 μL of the supernatant was transferred into HPLC vials containing 250 μL inserts.

Metabolic and lipidomic profiling was conducted using a Thermo Vanquish LC coupled to a Q Exactive HF Orbitrap mass spectrometer. Polar metabolites (cell extracts from aqueous phase) were measured using a previously described HILIC method [54], and 5 μL of the extracts was injected. Nonpolar metabolites (cell extracts from organic phase) were measured using a previously described RP method for lipid detection [54] with the adapted DDA parameters (AGC target of 1e6 and maximum injection time of 70 ms), and 5 μL of extracts was injected.

**Fig. 1:**
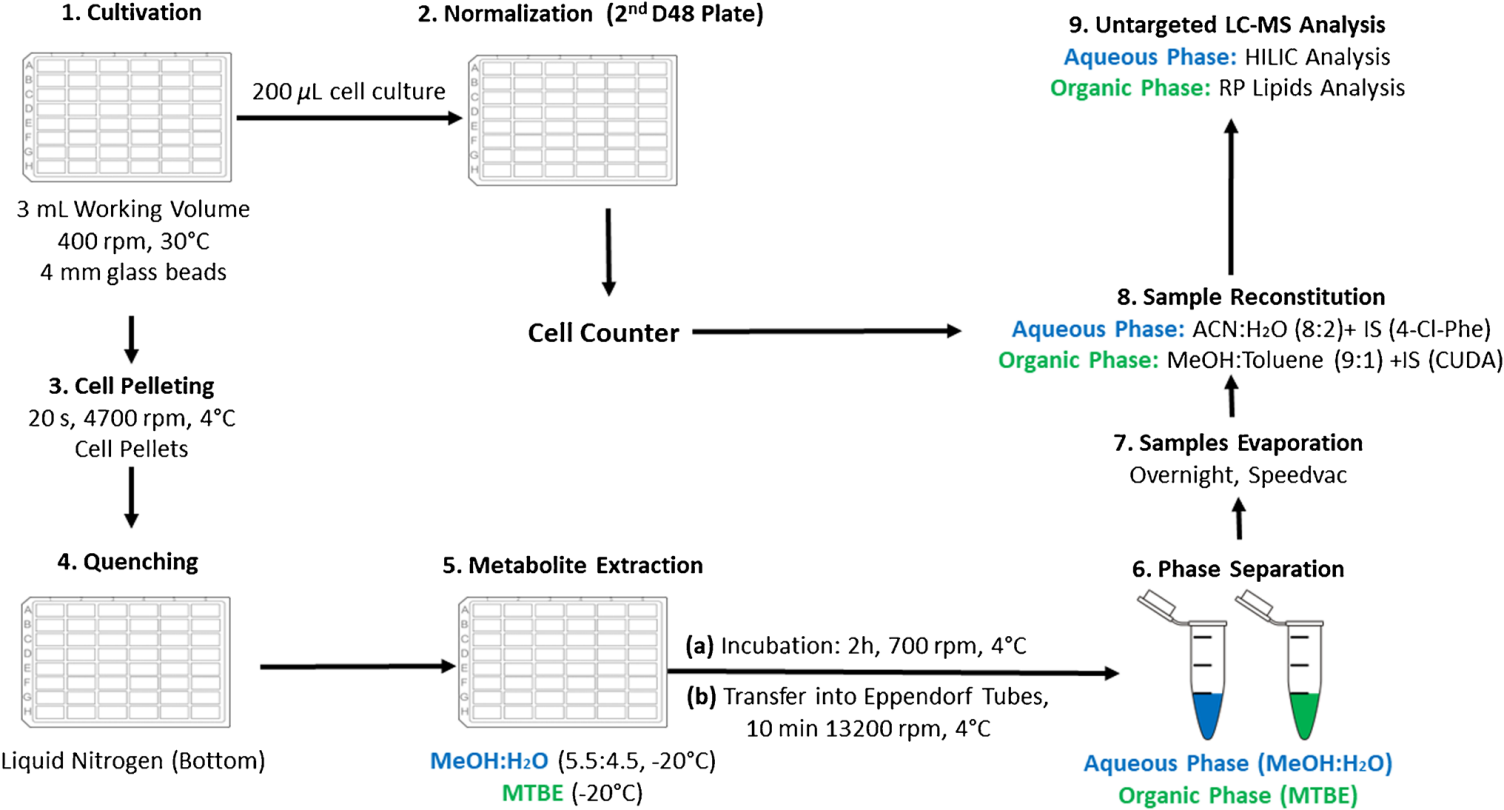
High-throughput sample generation using a D48-well plate. (1) Cultivation: yeast strains are cultivated in a D48 well plate in the presence of unlabelled and/or stable isotope-labelled substrates. (2) Normalization: before pelleting the cells, 200 μL aliquots of the cell cultures are transferred into a new D48 plate containing ISOTONE II (Beckman Coulter) solution for biovolume (μL/mL) determination using the Multisizer Z3 (Beckman Coulter). The latter is used to calculate the resuspension volume for the dried metabolite extracts prior to LC-MS analysis. (3) Cell pelleting: fast centrifugation treatment. (4) Quenching: the bottom of the plate is immerged in liquid nitrogen. (5) Metabolite extraction: the cell pellets are resuspended in pre-cooled MeOH:H_2_O (− 20 °C). After resuspension,  − 20 °C pre-cooled MTBE is added for metabolite extraction. After the incubation step, the cell extracts of each well are transferred into 2-mL Eppendorf tubes. (6) Phase separation: the cell extracts are centrifuged in order to achieve a phase separation between the upper organic phase (MTBE) and the lower aqueous phase (MeOH:H_2_O). (7) Sample evaporation: the collected phases are evaporated overnight using a SpeedVac vacuum device (Labconco). (8) Sample reconstitution: samples are reconstituted, adapting the resuspension volume to the biovolume (μL/mL) for the purpose of normalization. (9) Untargeted LC-MS analysis: the biovolume-normalized samples are analysed by HILIC-MS (metabolomics) or RP-LC-MS (lipidomics). Abbreviations are reported in the abbreviation list

A schematic representation of the experimental approach and techniques used in this study is summarized in Fig. 1.

#### Intracellular succinate quantification

Intracellular ^13^C succinate concentrations were quantified in uniformly ^13^C-labelled cell extracts with a newly designed method based on spiking with unlabelled (^12^C) succinate. The spike concentration was estimated using the measured intracellular ^13^C succinic acid areas and an external calibration curve generated with unlabelled succinate.

### Data analysis

The parameter settings for the different software described below (Proteowizard, MS-DIAL, PAVE, Shinyscreen, SIRIUS CSI:FingerID) are all available in the electronic supplementary material (ESM, Section S3).

#### Computational workflow

Raw LC-HRMS files of all samples (including extraction and procedural blanks) were first imported into MS-DIAL 4.8 [6] for peak detection, deconvolution, and alignment. Through this common peak-picking alignment input, each detected feature became a unique numeric MS-DIAL ID that was used to identify features in the subsequent data analysis. This feature list was imported into the PAVE 2.0 MATLAB GUI [56] using MATLAB version R2017b. Raw files were converted to mzXML files (Proteowizard, v3.0.20022-e71f69e07, [57]) and parsed to generate a single M file for each experimental setup, strain/condition, and ionization mode (.mat format, *n* = 4 for positive ionization mode, *n* = 4 for negative ionization mode, available in the ESM, GNPS [58]). The credentialing data analysis followed the PAVE workflow [41] and was performed separately for each strain and experimental condition tested using the generated M files (SF-WT, SF-KO, D48-WT, and D48-KO), and a list of credentialed features for each strain and condition was generated (ESM, Zenodo, files F03-04 [59]). The MS-DIAL ID was used to eliminate duplicate features by merging the credentialed features obtained for the WT and KO strains in each experimental setup after the credentialing analysis of PAVE. This generated list, containing unique features per experimental setup (referred to as “Total Features Exp. Setup” by the further steps of the data analysis), was used as the input for the next step of the computational pipeline (available by the ESM, Zenodo, files F05-08 [59]).

#### Quality control of credentialed features using Shinyscreen

The feature list fed into PAVE and the resultant carbon and nitrogen count of credentialed features was used to calculate the theoretical ^13^C^15^N-*m/z* for each feature. The calculated ^13^C^15^N-mass shift was used to perform a credentialing quality control using Shinyscreen (v1.0.3 [49]). Converted mzXML ^13^C^15^N files and the merged PAVE results coming from the same experimental setup (e.g. D48-WT-KO and SF-WT-KO, “Total Features Exp. Setup”) were used as the input. Features that showed the corresponding mass shift in the uniformly labelled data were retained (recognized by Shinyscreen and tagged with MS1 = TRUE, with results and data analysis steps available by ESM, Zenodo, files F05-08 [59]) and used for feature annotation and further data analysis.

#### Feature annotation

For the HILIC-based analysis, the feature annotation was performed using a confidence level scheme [60]. Level 1 or confirmed structures were assigned to features having a RT, *m/z*, and MS^2^ match with authenticated reference standards; Level 2A or probable structure was assigned by MS^2^ spectral matching using spectral databases; Level 3 or tentative structure candidates were obtained with the detected spectral information and predicted with in silico fragmentation tools; Level 4 or unequivocal chemical formula assignment was assigned using exact masses and natural isotope distributions; and Level 5 or mass of interest was assigned to features where the estimation of chemical composition or structure elucidation was not possible using the experimental data. The annotation was conducted by applying a hierarchical step-by-step approach using the cheminformatics software outlined below.

#### Annotation with MS-DIAL

First, features were putatively annotated in MS-DIAL as Level 2A following manual review if they fulfilled the minimal criteria of a dot product  ≥ 50% and fragment presence  ≥ 50%. These features were reported as “Level 2A MS-DIAL”. The database used for feature annotation of the HILIC data was the MSMS-Public-(Neg/Pos)-VS15.msp (available on the MS-DIAL website [6]).

#### Pre-screening and MS^2^ spectra extraction

The remaining features underwent the pre-screening with Shinyscreen [50] for MS^1^/MS^2^ alignment verification and MS^2^ extraction. Only features that passed this quality control step were used for further annotation (ESM, GNPS [58]).

#### Annotation with MetFrag and PubChemLite

The spectral information of features that passed the pre-screening with Shinyscreen were imported in MetFrag to achieve tentative identification using an early version of PubChemLite (PCLite, PubChemLite tier1 [61]). The R script for MetFrag is available on Zenodo (see ESM, Zenodo, file F10 [59]). To simplify the annotation, the spectral information of the samples showing the highest MetFrag overall score was used. Further, the carbon and nitrogen counts obtained with PAVE were compared with the molecular composition of the MetFrag-PCLite candidates. The correspondence of the number of carbon and nitrogen atoms between the PAVE and MetFrag-PCLite results allowed to annotate the features either as Level 2A (if a MoNA score  ≥ 90% was present) or as Level 3 and reported as “Level 2A MetFrag” or “Level 3 MetFrag”, respectively. The best three MetFrag Level 3 candidates were reported, together with the presence of annotation information from the “Interactions and Pathways” section, which indicates whether candidates may be of biological relevance [11].

#### Annotation with SIRIUS CSI:FingerID

The spectral information of the features remaining without annotation were imported into SIRIUS CSI:FingerID [13, 14]. Adapted software parameters were used, and all possible ionization adducts for the positive and negative modes were considered. The carbon and nitrogen number calculated by PAVE was specified in the CSI:FingerID parameters (see ESM, Section S3) where all the available databases and default adducts were used. Putative results were annotated as Level 3 when a possible structure candidate was found or as Level 4 in case of an unequivocal chemical formula match and reported as “Level 3 SIRIUS” or “Level 4 SIRIUS”, respectively.

#### Unknown features

The remaining orphan features were annotated as “Level 5” with the PAVE-calculated carbon and nitrogen numbers.

#### Manual quality control of credentialed features

**Fig. 2:**
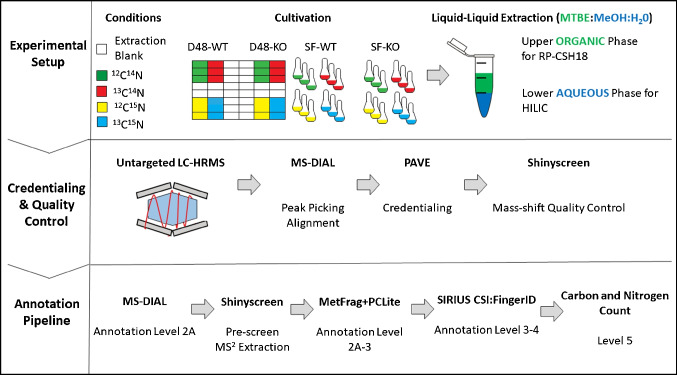
Experimental and computational pipelines used in this study. Yeast WT (ho:kanMX) and KO (sdh1Δ) strains were cultivated in the presence of unlabelled and/or labelled substrates in a D48 well plate and SF, followed by extraction of polar and nonpolar metabolites for LC-HRMS analysis. The raw data obtained was processed with the MS-DIAL peak-picker to obtain a feature list for the PAVE workflow. Credentialed features underwent a mass shift quality control using Shinyscreen. Features with confirmed mass shift were imported into MS-DIAL for Level 2A annotation. The MS^2^ spectra of the remaining, non-annotated features were extracted with Shinyscreen and annotated as Levels 2A–3 with MetFrag combined with the PubChemLite (PCLite) chemical database, or as Levels 3–4 with Sirius CSI:FingerID. The non-annotated features are reported as Level 5 with their relative carbon and nitrogen number. Abbreviations are reported in the abbreviation list

To estimate the quality of the computational pipeline results, a manual quality control step was conducted for the features that passed the above presented computational workflow using the Xcalibur software (Qual Browser, Thermo Fisher Scientific). Here, the carbon and nitrogen count information obtained with PAVE was used to calculate the mass shift in the ^13^C, ^15^N, and uniformly labelled condition (^13^C^15^N) of each feature. Features for which a corresponding unlabelled/uniformly labelled mass shift could not be confirmed upon this manual inspection were discarded and reported as false-positive credentialed features. The percentage was calculated as the ratio between false-positive credentialed features to the total amount of features which passed the computational pipeline. A schematic representation of the experimental setup and data analysis pipeline is shown in Fig. 2.

#### Lipid analysis

Only the first annotation step using MS-DIAL and the subsequently mass shift quality control with Shinyscreen were applied for the lipidomic analysis. The credentialed and curated lipid features were imported in MS-DIAL and putatively annotated as “Level 3 MS-DIAL” by a dot product  ≥ 40% [54]. Less strict annotation criteria compared to the HILIC data were used, since only an in silico spectral database was used for annotation.

#### Level 1 metabolite identification

Authentic reference standards were used to achieve Level 1 identification using an RT matching window of ± 0.2 min, a mass accuracy ± 10 ppm, and MS^2^ spectral matching. The identification results are provided in the supplementary files (see ESM, Zenodo, file F13 [59]).

#### Data visualization and statistical analysis

Data visualization and statistical analysis were only applied to the metabolomics (not lipidomics) data, where credentialed peak heights were normalized to the IS (4-chloro-L-phenylalanine). The normalized data were uploaded to MetaboAnalyst 5.0 and Pareto-scaled prior to principal component analysis (PCA). Excel was used to calculate the coefficient of variance (defined as ratio between standard deviation and signal intensity average, CV %) distribution between the two approaches and generate histograms. A one-way analysis of variance (ANOVA) followed by FDR-corrected and Tukey’s HSD post hoc tests (*p* value  < 0.01) was conducted in R to compare metabolic changes between groups. The results of the statistical analysis are available in the supplementary files (see ESM, Zenodo, file F12 [59]).

## Results and discussion

In this part of the study, we aimed to benchmark our proposed high-throughput approach for yeast cultivation and sample generation for credentialing-based untargeted metabolomics against a classical shake flask (SF) approach, by comparing physiological parameters such as growth and glucose uptake rates and intracellular succinate concentration, as well as credentialing and annotation results. Furthermore, we discuss the distribution of CV values and investigated metabolic differences between the analysed WT and KO strains using a parametric ANOVA test (Turkey’s HSD).

### Physiological constraints and intracellular succinate concentration

**Fig. 3:**
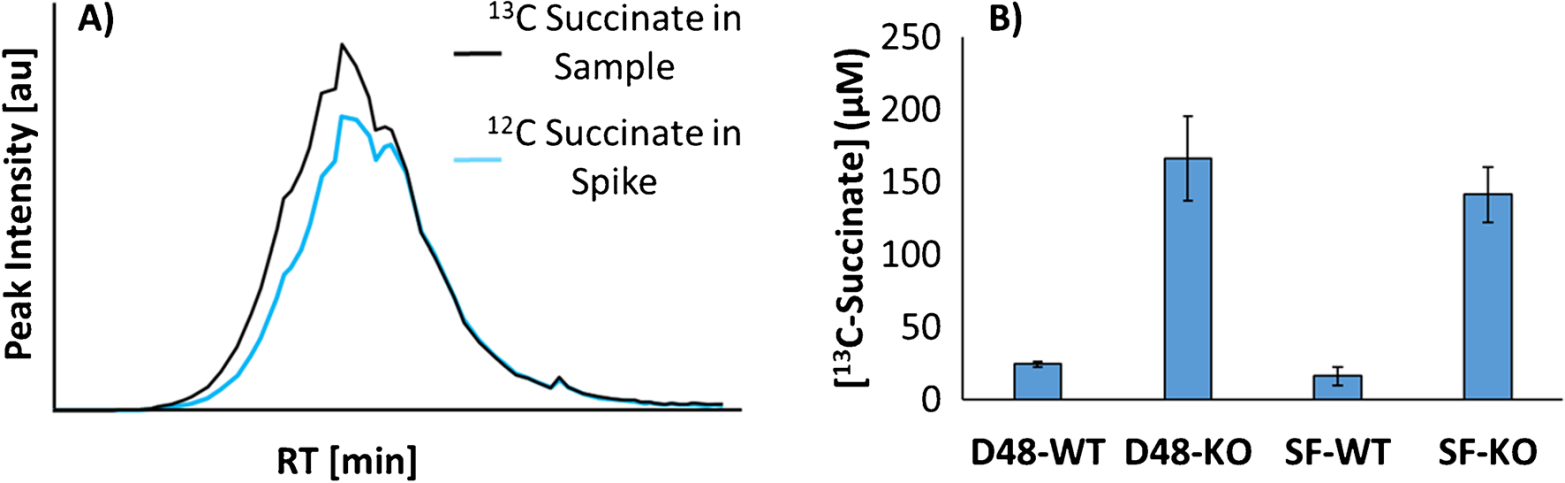
Quantification of intracellular ^13^C-Succinate in yeast WT and KO strains following classical SF or D48 cultivation. **A** The ^13^C uniformly labelled cell extracts (black, MS^1^ signal) were spiked with unlabelled succinate (light blue, MS^1^ signal). **B** Calculated intracellular concentration of ^13^C succinate for the yeast WT and KO strains after applying the D48 (D48-KO/WT) or SF (SF-KO/WT) approaches. The bar plot values refer to means ± SDs for three biological replicates. The ^13^C succinate concentrations calculated in μM amount to 24.5 ± 2.0 (D48-WT), 16.2 ± 6.3 (SF-WT), 166.0 ± 29.2 (D48-KO), and 141.3 ± 19.0 ( SF-KO). The resulting fold changes (KO/WT) were 7.2 ± 0.2 (D48) and 9.0 ± 0.9 (SF). Abbreviations are reported in the abbreviations list

The growth and glucose uptake rates were monitored in the control yeast strain cultivated in D48 and SF format. Similar linear ranges of the growth rate (0.37 and 0.36 h^−1^, respectively) and glucose consumption (4.17 and 3.45 mM∙h^−1^, respectively) were observed in both cultivation modes (ESM, Figures S1–3). In the SF condition, cells entered the glucose consumption phase earlier than in the D48 condition. We speculate that fine differences at the level of gene expression or other regulatory processes govern the entry into the high glucose uptake phase slightly differently in both experimental setups. Recently, deep-well well cultivation of *Pseudomonas putida* and *Pseudomonas aeruginosa* showed no remarkable differences in substrate assimilation compared to the SF approach, suggesting that deep-well based high-throughput methods represent a robust and flexible technique for performing microbial metabolic profiling [45]. The highly comparable bioprocess parameters obtained here suggest indeed that budding yeast, likewise, features very similar metabolic behaviour in deep-well cultivation as in the classical shaking flask format. Next, we cultivated a yeast strain deficient in the *SDH1* gene (KO), encoding the FAD-binding subunit of succinate dehydrogenase, and the corresponding control strain (WT) under SIL in the D48 and SF setups, and measured the intracellular succinate concentration using an innovative quantification approach based on spiking the ^13^C-labelled cell extracts with standard ^12^C-succinate. We observed the expected increase in the intracellular succinate concentration in the KO compared to the WT strain and the calculated KO/WT -fold change (FC) values were comparable with previously reported values [44] (see ESM, Zenodo, file F01 [59]). These results, summarized in Fig. 3, suggest that our D48 method should yield metabolomic results consistent with the classical SF approach. Moreover, by applying the proposed quantification method, we showed that the uniformly labelled cell extracts can be used in a versatile way for the quantification of intracellular metabolite concentrations, allowing for reduced experimental costs by circumventing the need for expensive labelled standards.

### Credentialing results

The data generated from the (un)labelled samples for each strain and experimental setup were processed individually with the PAVE software [41]. One reason for choosing the PAVE approach was to minimize dilution of low-abundant intracellular signals that may result from mixing unlabelled and labelled cell extracts as performed in other credentialing approaches [36, 40]. Tables 1 and 2 summarize the results of the credentialing analysis for the metabolomics data acquired in the positive and negative ionization modes, describing the number of discarded features (e.g. adducts, background peaks, dimers, fragments, heterodimers, isotopes, low carbon counts, low scores for chemical formula assignment with Pearson’s correlation coefficient  < 0.5, multicharges) and retained credentialed features [41]. We also report the total number of unique credentialed features detected per experimental setup obtained after merging the credentialed feature lists for the WT and KO strains by the different experimental setups (i.e. SF and D48) and removing duplicates. Duplicate entries were removed using the initial feature list generated by the MS-DIAL peak-picker. Prior to the credentialing analysis using PAVE, all the analysed samples (unlabelled condition for WT and KO for the SF and D48 approach, including extraction and procedurals blanks), were processed using MS-DIAL, and a list of features resulting from this common alignment was generated. This common alignment enabled the assignment of a unique numeric ID to each feature using MS-DIAL. The common alignment feature list was used as input for the PAVE data analysis in MATLAB, and the generated credentialed features conserved the numeric ID assigned using MS-DIAL. This unique numeric ID was used to eliminate duplicates value by merging the credentialing results outcoming from the different strains but from the same experimental setups. These total unique credentialed features list per experimental setup underwent the automated credentialing quality control check using Shinyscreen (filtering for features showing the expected mass shift in the uniformly labelled ^13^C^15^N cultivation conditions). The resulting retained features were used for the subsequent steps in our data analysis pipeline and for further comparison of the D48 and SF methods.

**Table 1:** Feature statistics following the PAVE-supported analysis of the HILIC-HRMS data acquired in positive ionization mode

PAVE output	HILIC-HRMS, positive ionization mode			
	D48-WT	D48-KO	SF-WT	SF-KO
Total detected features	16,518			
Adduct	666	532	572	591
Background	12962	13931	14138	13981
Dimer	22	13	14	16
Fragment	22	43	58	79
Heterodimer	36	31	24	27
Isotope	142	115	118	116
Low carbon count	95	84	65	61
Low score	759	440	256	301
Multicharge	53	30	21	27
Discarded features	14757	15219	15266	15199
Discarded features (%)	89.3%	92.1%	92.4%	92%
Credentialed features	1761	1299	1252	1319
Credentialed features (%)	10.7%	7.9%	7.6%	8%
Total unique credentialed features per setup	2077		1656	
Total unique credentialed features per setup (% vs. total detected features)	12.6%		10.0%	
Retained features*	**1115**		**908**	
***Retained features (%)***	**6.8%**		**5.5%**	

*Credentialed features that passed mass shift quality control criteria in Shinyscreen (i.e. showing the expected mass shift in the uniformly labelled ^13^C^15^N cultivation condition). Bold font indicates retained features that were used in the following workflow steps

**Table 2:** Feature statistics following the PAVE-supported analysis of the HILIC-HRMS data acquired in negative ionization mode

PAVE output	HILIC-HRMS, negative ionization mode			
	D48-WT	D48-KO	SF-WT	SF-KO
Total detected features	19828			
Adduct	77	46	172	156
Background	18308	18384	18041	18108
Dimer	2	1	22	13
Fragment	40	13	152	96
Heterodimer	5	0	33	26
Isotope	94	42	135	123
Low carbon count	50	24	68	60
Low score	239	270	90	127
Multicharge	6	0	3	2
Discarded features	18821	18780	18716	18711
Discarded features (%)	94.9%	94.7%	94.4%	94.4%
Credentialed features	1007	1048	1112	1117
Credentialed features (%)	5.1%	5.3%	5.6%	5.6%
Total unique credentialed features per setup	1441		1400	
Total unique credentialed features per setup (% vs. total detected features)	7.3%		7.1%	
Retained features*	**1004**		**705**	
***Retained features (%)***	**5.1%**		**3.6%**	

*Credentialed features that passed mass shift quality control criteria in Shinyscreen (i.e. showing the expected mass shift in the uniformly labelled ^13^C^15^N cultivation conditions). Bold font indicates retained features that were used in the following workflow steps

The number of discarded (e.g. adducts, background) and credentialed features for the different strains and experimental conditions following the metabolomics analyses is highly comparable (Tables 1 and 2). Multiple processing attempts revealed that the best way to apply the PAVE approach and perform comparative metabolomics between WT and KO strains was to process the data from the different strains or conditions separately. We assume that processing WT and KO samples together proved to be problematic for the ATOMCOUNT function in PAVE which uses peak intensity (within a given threshold, e.g. applied threshold 0.5) as the criterion to identify credentialed features [41] and undoubtedly can be highly strain or condition dependent (e.g. genetic background, cultivation method, supplementations). Processing the strains and conditions separately increased the data analysis time but yielded comparable percentages of credentialed features to Wang and co-authors (between 3.1–4.6% [41] versus 5.1–6.8%, herein for the D48 experimental setup) who analysed single strains grown in a single condition, suggesting that the applied data processing method is suitable. Compared to Wang and co-authors, we used less stringent credentialing parameters (e.g. Pearson correlation coefficient cut-off of 0.75 by PAVE and 0.5 in this work; for complete parameter setting see ESM, Sect. 3) that was compensated for by our downstream quality control steps. This modification allowed us to better retain false negatives that were otherwise discarded by the software, which is particular useful for our practical application and research interest relative to metabolites mapping of canonical and non-canonical metabolites (the latter known to be less abundant and more challenging to detect). Ultimately, our workflow results in high-quality credentialed features (i.e. “retained features”) to use in our annotation approach. The percentage of credentialed features obtained via lipidomics for the nonpolar extracts using PAVE and Shinyscreen is reported in the ESM (Sect. 2, Tables S1–2).

### Results of feature annotation

#### Annotation of the credentialed yeast polar metabolome

Following feature credentialing with PAVE and quality control using Shinyscreen, we proceeded with our hierarchical step-by-step annotation approach. The first step matched features with the integrated MS^2^ spectral database in MS-DIAL, and the positively identified features (ESM, Zenodo, file F09 [59]) were labelled as Level 2A (MS^2^ spectral database matching). The remaining features were further processed with Shinyscreen to perform a pre-screening quality control step to verify MS^1^/MS^2^ alignment prior to MS^2^ spectral extraction. This spectral information was used for further analyses in MetFrag combined with PCLite or SIRIUS CSI:Finger ID. Finally, the quality of the credentialing/annotation results was checked manually by recovering the signals for the annotated features to determine the percentage of false-positive entries (i.e. that were credentialed via the automated workflow, but for which the expected mass shift in the fully labelled condition could not be confirmed manually). The processing and annotation results for credentialed polar metabolites are summarized in Tables 3 and 4.

**Table 3:** Pre-screening results of Shinyscreen for the HILIC-HRMS data acquired in positive and negative ionization modes. Features with an acceptable MS^1^/MS^2^ alignment underwent annotation with MetFrag-PCLite and SIRIUS CSI:FingerID

HILIC, positive ionization		Processed features	HILIC, negative ionization	
D48	SF		D48	SF
1115	908	Retained features	1004	705
110	106	Level 2A MS-DIAL	83	95
1005	802	Retained features (without level 2A MS-DIAL)	921	610
286	264	MS^1^/MS^2^ alignment	214	215
28.5%	32.9%	MS^1^/MS^2^ alignment (% vs. retained features)	23.2%	35.2%

**Table 4:** HILIC-HRMS-based annotation results for the credentialed features (positive and negative ionization modes) with their absolute value per experimental setup (abs. value) and relative percentage (%) to the features with an MS^1^/MS^2^ alignment. False-positive percentage refers to the ratio of the annotated features in which a mass shift could not be confirmed manually in the labelled raw data to the total amount of annotated features prior the mass shift quality control

HILIC, positive ionization				Total annotated features	HILIC, negative ionization			
D48		SF			D48		SF	
286		263			214		215	
Abs. value	[%]	Abs. value	[%]	Confidence level	Abs. value	[%]	Abs. value	[%]
110	38.5	106	40.2	Level 2A MS-DIAL	83	38.8	95	44.2
5	1.7	3	1.1	Level 2A MetFrag	0	0	2	0.9
107	37.4	96	36.4	Level 3 MetFrag	50	23.4	46	21.4
11	3.8	9	3.4	Level 3 SIRIUS CSI:FingerID	30	14	19	8.8
11	3.8	9	3.4	Level 4 SIRIUS CSI:FingerID	8	3.7	5	2.3
42	14.7	40	15.2	Level 5	43	20.1	48	22.3
8%		7%		False-positive (no-mass shift/total annotated)	15.4%		12.6%	

The aim of this work was to compare the credentialing and annotation performance in our developed high-throughput D48-well approach with the low-throughput, classical SF cultivation format, in addition to showing a potentially application of credentialing in the D48 approach. With the goal to generate a list of annotated features, in an automated and unbiased way, to use the resulting feature lists as a metric for method comparison, the credentialed features were annotated by applying defined rules. With MS-DIAL, for instance, a Level 2A was assigned based on parameters such as a minimum dot product of 50% and a fragment presence (i.e. irrespective of intensity) of 50% by comparison of the experimental results with a freely available MS^2^ spectral database. We did not modify the annotation results and included duplicate annotation entries (e.g. isobars with different RT time and fulfilling the criteria for MS^2^ spectral match with the reference MS^2^ experimental database) and less likely biological molecules or potential fragment molecules that were not successfully discarded by PAVE (e.g. MS-DIAL ID 1190 [M+H]^+^, 3-methylpyrazole, PubChem CID 15073; MS-DIAL ID 1366 [M+H]^+^, morpholine, PubChem CID 8083). As summarized in Table 3, the total amount of annotated features that passed the final manually curated mass shift quality control step (286 and 264 in the positive ionization mode, for the D48 and SF samples, respectively; 214 and 215 in the negative ionization mode, for the D48 and SF samples, respectively) was lower compared to the number of credentialed features that had passed the mass shift quality control with Shinyscreen (1115 and 908 in the positive ionization mode, for the D48 and SF samples, respectively; 1004 and 705 in the negative ionization mode, for the D48 and SF samples, respectively) and referred to as “Retained features” in Tables 1, 2, and 3. This is due the fact that the MS data acquisition was performed in DDA mode and only credentialed features with an MS^1^ aligned with detected MS^2^ events were used for the annotation (obtained with Shinyscreen and referring to “MS^1^/MS^2^ alignment” in Table 3) and some of the features that passed the automatic mass shift quality control with Shinyscreen were erroneously retained. We report these erroneously retained features and explain these as the overall false-positive entry generated by the proposed computational pipeline. Our results underlie the fact that often a combination of different software could be challenging in the praxis and parameters setting and especially fine tuning of these affect the outcome of an untargeted analysis. In this works, we aim to show and propose a way to combine open-source software and conduct metabolomics data analysis without coding knowledge using community-developed software with user-friendly interfaces. Furthermore, we would like to show the value of credentialing information, which enables us to validate the biological origin of features and inevitably allows us to confidently answer biological questions. Despite this, the annotation results, summarized in Table 4, led to a comparable number of annotated features per confidence level for the D48 and SF experimental setups, in both ionization modes, with also comparable false-positive percentages, determined after the final manual mass shift quality control. To assess the biological relevance of the annotation results, we performed an InChIKey-based search against the Yeast Metabolome Database (YMDB) [18, 19] and the Human Metabolome Database (HMDB) [16, 17]. For this, we used the InChIKeys obtained by the MS-DIAL annotation (Level 2A MS-DIAL) and generated an InChIKey list without duplicates entries. The number of exact InChIKey matches and of exact InChIKey first block matches between the annotated HILIC-HRMS features and both databases is shown in Table 5. A full analysis is available in the ESM (Zenodo, file F14 [59]).

**Table 5:** InChIkey recovery comparing YMDB and HMDB with the annotated features from the HILIC-HRMS data analysis. “POS” and “NEG” refer to the positive and negative ionization modes, respectively. Absolute values (Abs. value) and percentage matches to the total unique Level 2A annotated features per experimental setup are reported

Unique Level 2A MS-DIAL per experimental setup	Level 2A MS-DIAL POS/NEG			
	D48		SF	
	112		115	
InChIKey match	Abs. value	[%]	Abs. value	[%]
Exact InChIKey match in YMDB	31	27.4	30	26.1
Exact InChIKey First Block (skeleton) match in YMDB	81	71.7	83	72.2
Exact InChIKey match in HMDB	44	38.9	42	36.5
Exact InChIKey First Block (skeleton) match in HMDB	101	89.4	103	89.6

The InChIKey-based database search also revealed highly comparable numbers of database matches between the D48 and SF experimental setups for both ionization modes (Table 5). All this further indicated that the D48 cultivation and sample generation approach represents a solid basis for unbiased metabolite mapping in yeast. Intriguingly, the HMDB-based database search resulted in more matches than YMDB (Table 5). This suggests that potentially more of the metabolites reported in HMDB (220,945 small molecule entries, last updated 2022) are to be found in yeast, although not yet reported in YMDB (16,042 small molecule entries, last updated 2017). The HMBD annotation results that are not present in YMDB would still have to be confirmed with authenticated chemical standards. However, the InChIKey-based database search results show how the choice of the chemical database used in a biological study may influence the biological interpretation of the resulting annotation, and the choice of the reference chemical database has to be considered when judging the annotation results in the context of a specific biological study. An important consideration was how our results compared to the ones of Wang et al. (2019), although a direct comparison was challenging due to differences in metabolite extraction and LC-MS methods, instrumentation, and metabolite confidence level reporting. To simplify the comparison, we decided to only compare the 500 features annotated in our D48 method with their 221 annotated features using the first block of the unique InChIKey entries. These were obtained by converting their metabolite list (found in their supplementary information under “Annotation of all peaks” in the filename “ac8b03132_si_004.xls”) to InChIKeys using the PubChem Identifier Exchange Service [62]. Of the 221 overall annotated metabolites by Wang et al. (2019), 136 unique InChIkeys were obtained, while 417 unique InChIKeys were obtained for the 500 features annotated in our work. Only 56 metabolites overlapped in the two studies, while 80 metabolites were unique to Wang et al. (2019) and 341 unique to our pipeline (results available in ESM, Zenodo, file F15 [59]). The major difference in our annotation workflow is that it expanded beyond Levels 1 and 2 annotation and included in silico fragmentation (Level 3 annotation) and unequivocal chemical formula assignment (Level 4 annotation, although this does not yield InChIKeys). The reported unknown features in PAVE were 22.1% and 30.3% (205 of 926 and 209 of 690 credentialed features for the positive and negative ionization modes, respectively) of the total credentialed features [41]. This is slightly higher than the Level 5 feature numbers reported in this study (Table 4, 14.27% and 20.1% Level 5 features of the total retained features with MS^1^/MS^2^ alignment in positive and negative ionization mode with the D48 method). Nevertheless, both studies highlight that many unknown metabolites remain to be elucidated in the yeast metabolome and credentialing represents an important approach for tackling this knowledge gap. As recently demonstrated, the additional spatial selectivity gained by coupling HRMS to ion mobility spectroscopy may further enhance credentialing efforts and annotation confidence [63]. Moreover, identification is not limited to exact mass and collecting biologically relevant MS^n^ spectra but also having the relevant chemical databases for annotation as shown by the discrepancy between YMDB, HMDB, and PubChemLite. Tools to predict metabolites resulting from enzymatic side activities [64], non-enzymatic chemical damage, and biotransformation reactions [65, 66] provide useful resources for expanding chemical databases beyond the known chemical space. However, it remains difficult to validate good candidates beyond in silico approaches if authenticated standards or MS^2^ spectra are not available. Nevertheless, the confidence in the biological origin of detected features gained through credentialing approaches provides motivation to pursue the identification of unknown or low-level peaks that would normally be discarded. Recently, SIL-based credentialing metabolomics analyses in erythrocytes infected with the malaria parasite *Plasmodium falciparum* provided the basis for comparison and identification of gaps within the metabolic model of the disease, where 41% of the metabolome predicted from the parasite’s genome was covered in their multi-method analysis of polar extracts with GC-MS and LC-MS and nonpolar extracts using LC-MS [67]. Importantly, their analysis revealed the existence of non-canonical (non-predicted) metabolites and aided enzyme function discovery, further illustrating the utility of credentialing as a tool in completing metabolic networks.

#### Potential of using credentialing to facilitate lipid annotation

Analogous to the annotation of polar metabolites, we applied our rule-based annotation approach to the lipid data. As the lipid data analysis was done mostly as a feasibility check without prior parameter optimization or further refinement of the algorithm, the summarized annotation results of the relatively low number of credentialed features retrieved are reported in the ESM (Sect. 2.1., Table S3) and not described further here. One example of credentialed lipid molecule, putatively annotated as 1-tetradecyl-2-acetyl-sn-glycero-3-phosphocholine (PC (O-16:0), is shown in Figure S4 with related mass shift across the labelled conditions and match between the experimental and predicted MS^2^ spectra.

Credentialing is a strategy that, to the best of our knowledge, has not been applied to assist in the annotation of lipids, yet it offers solutions for some challenges in the field, such as signal deconvolutions, elimination of in-source fragment signals or de-adducting (as highlighted in the PAVE workflow [41]), and the quantification of lipid species which is normally difficult due to lack of isotope-labelled chemical standards [68]. The confirmed biological origin together with chemical formula information obtained with credentialing (e.g. observed mass shift and related carbon/nitrogen counts) may assist with correct feature annotation and help to discriminate between different lipid candidates. Additionally, spiking ^13^C-labelled lipid extracts with non-labelled lipid standards could help improve intracellular lipid quantification and thus provide deeper insights into lipid metabolism dynamics or turnover occurring during biological processes or disease development [69–71]. The raw data from the RP-LC-HRMS analysis of (un)labelled nonpolar extracts derived from all cultivations tested in this study are available as an open access data set (see ESM, GNPS [58]).

### Feature variance in the D48 approach

**Fig. 4:**
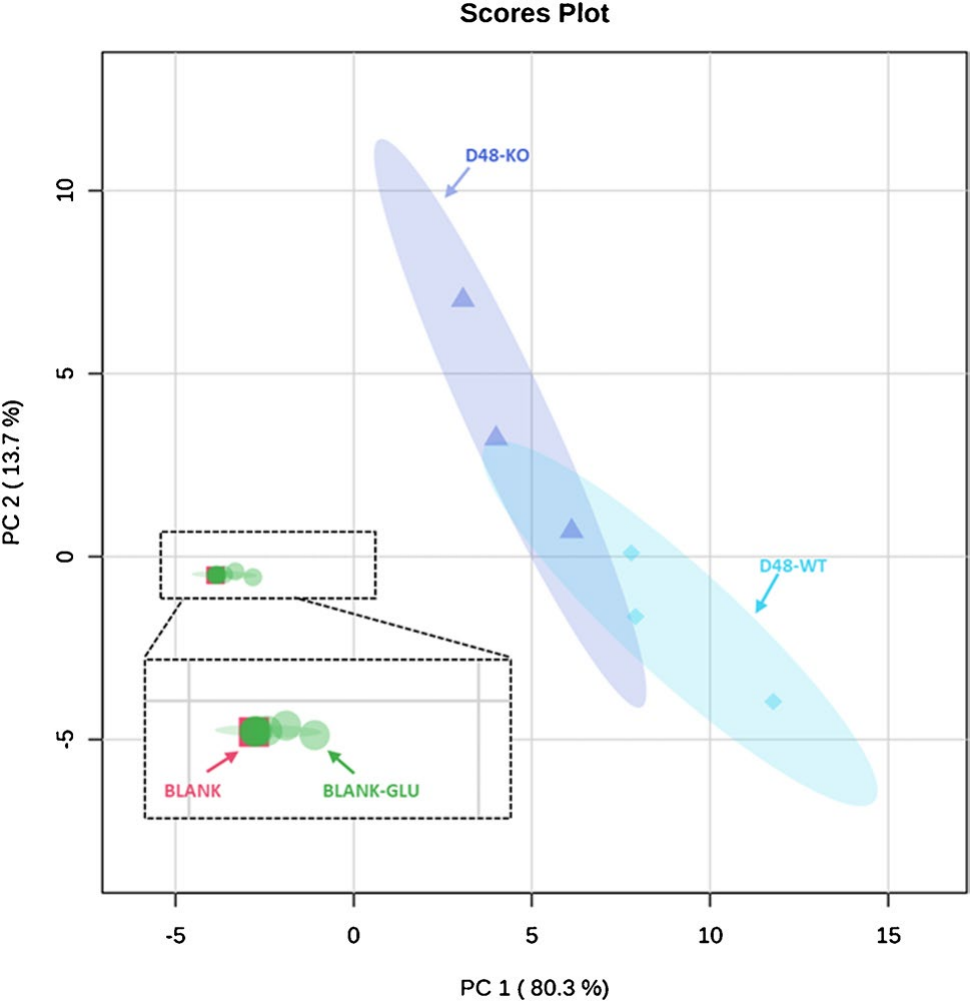
PCA score plot of the annotated credentialed features from the HILIC analysis of the D48 cultivation. Green dots: extraction blank samples (BLANK-GLU, *n* = 8). Red squares: procedural blank samples (BLANK, *n* = 3). The dashed zone shows the section of the PCA plot, where extraction and procedural blanks overlap. Purple triangles: KO samples from the D48 method (D48-KO, *n* = 3). Light blue diamonds: WT samples from the D48 method (D48-WT, *n* = 3)

A potential limitation with our D48 cultivation and extraction method is that it could introduce more experimental variance in comparison to the SF approach. First, we performed PCA to visualize the variance between D48 unlabelled metabolite extractions, extraction blanks, and procedural blanks. Procedural blanks (Fig. 4, “BLANK”) consisted of the resuspension solvent with IS, while extraction blanks (Fig. 4, BLANK-GLU) were samples generated from wells of the D48 plate containing ^12^C-YNB medium (with glucose) only that went through the entire experimental pipeline. Normalized peak intensities of the annotated features from both ionization modes of the HILIC-HRMS analyses for the D48 well format were imported to MetaboAnalyst 5.0 [48]. As shown in Fig. 4, a partial separation between the WT and KO samples is observed by principal component analysis. This partial separation seems in line with the results obtained in the quantitative approach used by Ewald et al. (2009) to compare intracellular concentrations of central carbon metabolites of the same yeast strains [44]. In this previous study, accumulation of the substrate (succinate) of the deleted enzyme was by far the most prominent difference observed between the *sdh1*Δ and the WT strains, while similar or indistinguishable levels were found for other central carbon metabolism intermediates. Procedural and extraction blanks grouped closely together, suggesting that their profiles are nearly identical. The clear separation between the blank and biological samples strongly suggests that there was negligible cross-over to other wells. Supporting this, the average normalized peak intensities of the biological and extraction blank samples were calculated and compared. From the total amount of analysed features (*n* = 500 times two strains, Σ = 1000), 956 showed fold change values  > 5 between biological and extracted ^12^C-YNB media blank samples (“BLANK-GLU”, median FC = 60 with 90^th^ percentile = 327.2), indicating that negligible cross-contamination between the wells occurred during cultivation and metabolite extraction.

**Fig. 5:**
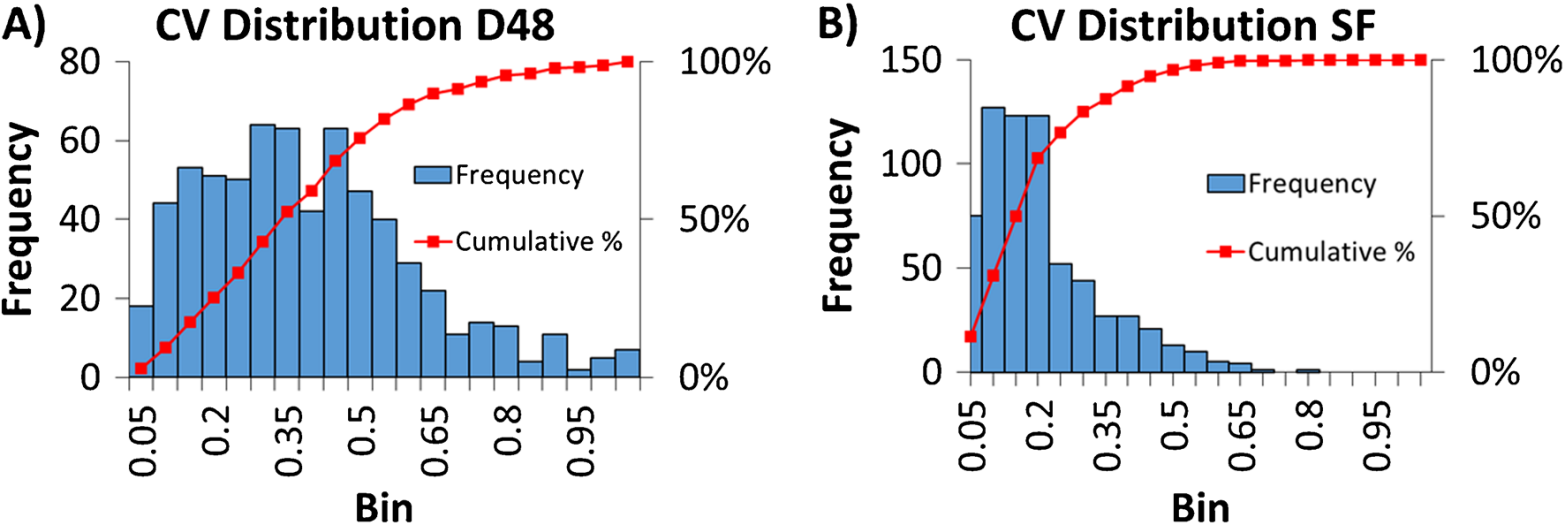
CV distribution for annotated credentialed features measured through the D48 (**A**) and SF (**B**) approaches. The bar plot represents the number of features inside 5% CV bin intervals, whereas the red line shows the cumulative frequency

Since PCA represents an exploratory data analysis method, we next examined the CV distribution of credentialed annotated features between the D48 and SF approaches to compare experimental variance. CV values should represent the total variability induced through all the steps of the experimental pipeline, and their distribution allows to assess the precision, reproducibility, and suitability of the experimental setups [40]. For each experimental setup, the CVs of the annotated features in both strains were calculated, and the distribution was split up into 5% bins and visualized with histograms (Fig. 5). The CV values for the D48 experimental setup (Fig. 5A) showed a wider distribution compared to the SF approach (Fig. 5B). The median CV value of the D48 setup was 34% with a 90th percentile of 66%, whereas the median CV value for the SF approach amounted to 15% with a 90th percentile of 39%. Comparing the D48 and SF approaches, 52.5% and 87.4% of the features, respectively, had lower CV values than 35% (see ESM, Zenodo, file F16 [59]). As observed by others before [44], this shows that using multi-well plate-based cultivation and extraction methods for increased throughput in microbial sample generation for metabolomics analyses comes at the price of higher errors and experimental variability. Specifically in our approach, we assume that the higher observed experimental variation was mainly introduced through the different pipetting steps using a multichannel pipette (sample normalization and metabolite extraction, see the “Materials and Methods” section) and less precise handling possibilities when working in a multiplex format compared to processing single samples individually, as also noted previously [44]. Blank subtractions or CV cut-off thresholds between 20 and 30% are commonly used in untargeted metabolomics data analysis pipelines for feature prioritization [72]. Applying a 35% CV cut-off in our data would discard approximately 50% and 13% of the features detected with the D48 and SF approaches, respectively. Prioritizing the subsequent annotation effort for a subset of features using a CV cut-off would improve the significance of the obtained results. However, by applying this strategy, low-abundant signals or features with higher variance due to very low intracellular concentration or non-specific detection, would not be annotated. This means that precious information about unknowns or non-canonical metabolites would not be considered further. Credentialing-based metabolomics data analysis does not depend on a posteriori statistical significance for feature prioritization. While the higher dispersion of the metabolomics data after D48 cultivation and sample generation can thus be overcome in combination with credentialing strategies for feature prioritization and may not greatly affect metabolite and pathway mapping in microorganisms, it nevertheless makes it more challenging to detect subtle metabolite level changes in comparative analyses between different strains or conditions than with classical, low-throughput approaches.

### Impact of genetic background and cultivation approach on metabolism

To explore the utility of the presented methods to investigate the metabolic impact of genetic alterations, the metabolic perturbations induced by succinate dehydrogenase deficiency in yeast were investigated based on the credentialed D48 and SF metabolomic datasets. A parametric ANOVA followed by FDR-corrected, Tukey’s HSD post hoc tests (*p* < 0.01) was performed in R using normalized peak height intensities of metabolites present in both experimental setups (*n* = 327; see ESM, Zenodo, file F16 [59]). We found that 52 or 15.9% of features showed statistically significant differences between the groups tested (D48-WT/KO and SF-WT/KO), with 32 or 9.8% of the total features showing an opposite WT versus KO trend in both experimental setups (ESM, Zenodo, file F16 [59]). Metabolite changes in the same direction between WT and KO in both experimental setups are interesting to consider as robust consequences of the genetic perturbation that are maintained in different environments. Metabolites showing opposite trends in WT versus KO in both experimental setups may be more affected by the different cultivation formats (D48 versus SF) than by the gene deletion.

**Fig. 6:**
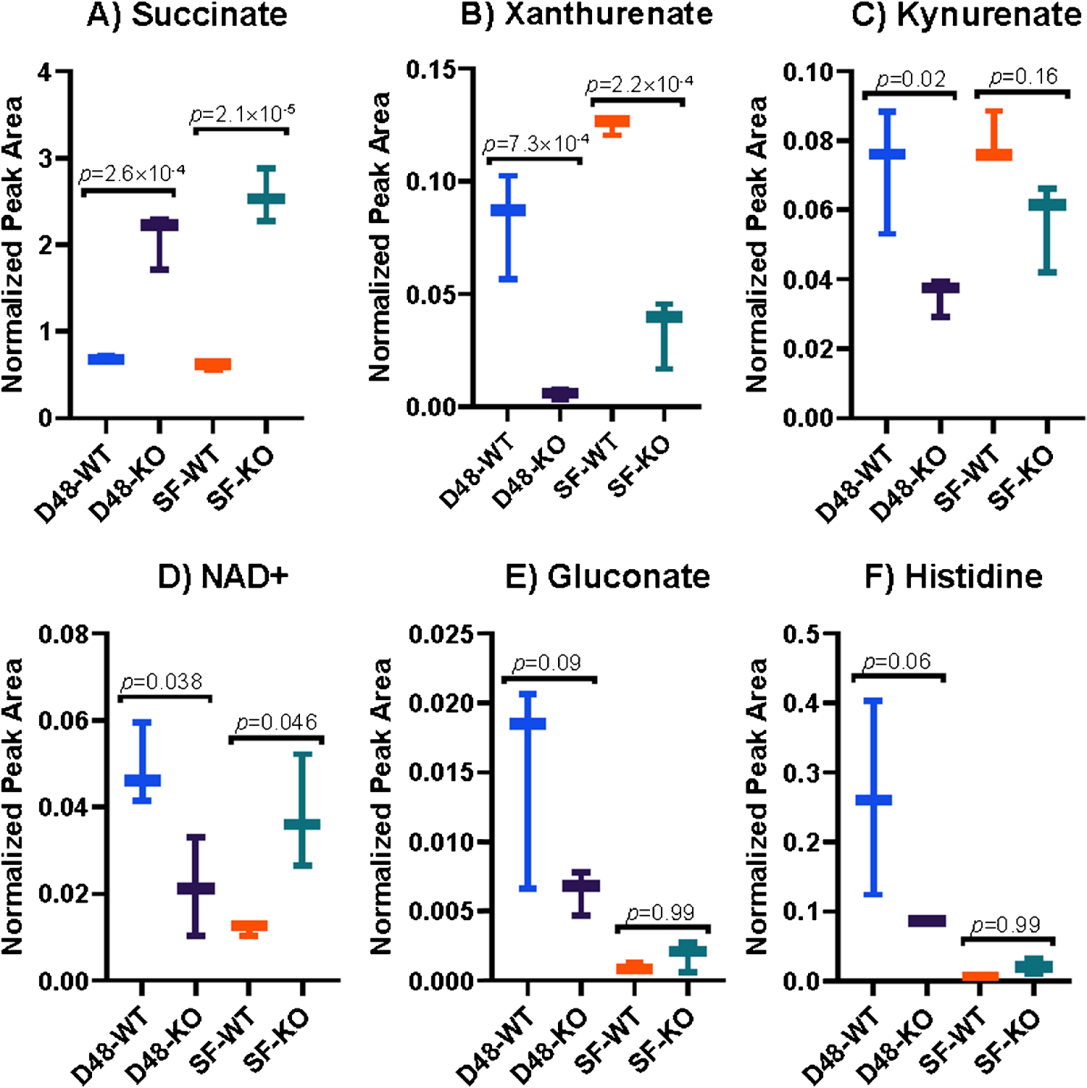
Box plot of the selected differential metabolites in D48 and SF conditions. Statistical significance was evaluated using a one-way ANOVA followed by Tukey’s HSD post hoc test (*p* < 0.01) from the HILIC-HRMS analyses. Metabolites were grouped by signals showing the same (succinate, xanthurenate, and kynurenate) or opposite (NAD^+^, gluconate, histidine) trends. The identities of succinate (**A**), xanthurenate (**B**), kynurenate (**C**), NAD^+^ (**D**), gluconate (**E**), and histidine (**F**) were confirmed (Level 1)

As described above and previously described by others [44], succinate levels were significantly higher (Fig. 6A; D48-WT vs. D48-KO *p* value = 2.61∙10^−4^, SF-WT vs. SF-KO *p* value = 2.08∙10^−5^) in the *sdh1*Δ strain compared to the WT strain in both approaches. The calculated FCs between KO and WT strains amount to 3.0 for the D48 and 4.3 for the SF approach. These values differ from the FCs calculated using the above presented quantification approach (FCs of 7.2 ± 0.2 and 9.0 ± 0.9 found with the D48 and SF samples, respectively), which are based on absolute concentrations instead of relative values (normalized peak height). The intracellular succinate concentration change is the most proximal effect expected from the gene deletion, as succinate is the substrate of the enzyme deficient in the analysed KO strain. Interestingly, we observed that xanthurenate showed the exact opposite trend to succinate, with depleted levels in the KO strain compared to the WT strain in both experimental setups (Fig. 6B; D48-KO vs. D48-WT: *p* = 7.26∙10^−4^, FC = 0.27; SF-KO vs. SF-WT: *p* = 2.2∙10^−4^, FC = 0.27). Xanthurenate is formed through transamination of the tryptophan catabolic pathway (or kynurenine pathway) intermediate hydroxykynurenate [73]. Kynurenate, another kynurenine pathway derivative [74], showed also the exact opposite trend to succinate with decreased levels in the KO strain compared to the WT strain, but this feature only showed statistical significance in the ANOVA (overall *p* = *0*.008) with post hoc tests failing to show significance for the SF samples (Fig. 6C; D48-KO vs. D48-WT: *p* = 0.02, FC = 0.49; SF-KO vs. SF-WT: *p* = 0.16, FC = 0.71). The observed differences in the levels of succinate, xanthurenate, and kynurenate are intriguing, since succinate dehydrogenase deficiency can cause the development of rare neuroendocrine tumours (e.g. bladder tumours [75]) and succinate ranks amongst the known oncometabolites [76, 77]. Furthermore, perturbations in tryptophan metabolism and increased excretion of tryptophan intermediates were observed in mouse models and patients with bladder cancer [78]. This preliminary data demonstrates a potential enhanced excretion of xanthurenate and kynurenate by the *sdh1*Δ KO compared to the WT. Further experiments are needed to validate these preliminary findings; however, they may support a link between succinate dehydrogenase deficiency and perturbation in tryptophan catabolism that could be useful to explore for cancer research. In this regard, our metabolomic dataset may represent an interesting resource for uncovering conserved metabolic perturbations caused by succinate accumulation that may potentially contribute to tumorigenesis, but further confirmation is needed for corroborating this hypothesis. Potentially, the quantification strategy proposed in this work, based on the use of non-labelled standards in fully labelled extracts (see “Material and methods”, subsection *Intracellular succinate quantification*), could be applied for future targeted studies that aim to quantify a potential relationship between *SDH1* deficiency and perturbation of tryptophan metabolism at reduced costs. As the kynurenine pathway leads to de novo nicotinamide adenine dinucleotide (NAD^+^) synthesis [74], we looked into the levels of this cofactor in our credentialed metabolomic dataset. Notably, a feature having the expected *m/z* value for NAD^+^ and the same RT as standard NAD^+^ was discarded as a background signal with PAVE in the D48 samples but successfully credentialed and annotated as NAD^+^ in the SF samples. The expected NAD^+^ mass shift was manually confirmed by comparing unlabelled and labelled data from both the D48 and SF samples, exemplifying that going back to the (un)labelled raw data may help retrieving false-negative signals and enhance feature annotation. The observed intracellular NAD^+^ signals showed about twofold changes between WT and KO strains, but with opposite directionalities in the D48 and SF samples (Fig. 6D), thus indicating that the effect of SDH deficiency on NAD^+^ levels is strongly dependent on the cultivation format. Furthermore, gluconate showed a decreased signal in the KO strain compared to the WT strain using the D48 approach (FC = 0.42), whereas in the SF approach, the gluconate signals showed comparable levels in both strains (Fig. 6E; ANOVA *p* value = 0.007; post hoc tests failed to show significance). Gluconate can be derived from the pentose phosphate pathway by dephosphorylation of 6-phosphogluconate [79] and higher gluconate levels could indicate that the pentose phosphate pathway has an enhanced activity in the D48-WT group compared to the other biological groups. Supporting this assumption, the intracellular histidine signal showed the same intensity level pattern and WT vs. KO trend as the gluconate signal (Fig. 6F; ANOVA *p* value = 9.0∙10^−3^). Since phosphoribosyl pyrophosphate is produced from the pentose phosphate pathway intermediate ribose-5-phosphate and is a precursor of histidine [80] (both not detected or annotated by the applied LC-MS method and annotation pipeline), an enhanced pentose phosphate pathway activity could provide more phosphoribosyl pyrophosphate, resulting in higher histidine biosynthesis, as previously observed in biotechnological optimized microorganisms [81]. The reason behind the higher gluconate levels in the D48-WT group compared to all other groups remains currently unknown.

## Conclusions

In this work, we combined a credentialing method (PAVE approach [41]) based on stable isotope labelling with high-throughput yeast cultivation (D48) and extraction to perform untargeted metabolomics using LC-HRMS, followed by an elaborate pipeline of quality control steps and compound annotation tools and finally, manual curation steps. Growth and glucose uptake rates of the high-throughput yeast cultures were highly comparable to the ones of classical SF cultures. We proposed a new intracellular quantification strategy, by spiking ^13^C-labelled cell extracts with non-labelled standard, a method that does not depend on using expensive labelled chemical standards. We adapted our cultivation method from Ewald and co-authors [44] and used the yeast *sdh1*Δ strain as well as a wild-type control strain for comparative metabolomics analyses. Data processing with our adapted PAVE workflow led to a comparable amount of credentialed features between the D48 and classical SF approach, and the subsequent annotation, performed using open data sources (MS-DIAL, MetFrag, SIRIUS CSI:FingerID [6, 12–14]), led to a comparable amount of annotated features. We found that the optimal way to perform a credentialing analysis using PAVE with different yeast strains is to process the different biological groups or conditions separately, despite the associated increase in processing time. For future works, integrated, tailor-made computational tools could support the development of a semi-automated annotation pipeline to reduce the overall analysis time. The high-throughput sample generation method described here enables faster sampling and metabolite extraction compared to a classical SF approach. However, this comes at the cost of increased experimental variance for the credentialed features due probably in large part to increased experimental errors when working in multiplex format, increasing the importance of subsequent targeted validation experiments. Nevertheless, using the D48 approach, we detected 2119 credentialed features (5.8% out of 36346 features detected in total in positive and negative modes) across the analysed strains and of those we were able to annotate 198 with high confidence (MS^2^ database match), showing the potential of the D48 method for comparing high number of true biological signals between different strains and/or conditions in one single experiment. The ANOVA between the different strains and conditions tested revealed that 52 or 15.9% of the total analysed features (*n* = 327) showed statistically significant differences amongst the groups (D48-WT/KO and SF-WT/KO), with 32 or 9.8% of the features showing an opposite WT versus KO trend in both experimental setups and 20 or 6.1% of the features showing changes with the same directionality (see ESM, Zenodo, file F16 [59] for details). Preliminary analyses also suggest that our pipeline can be further developed to perform credentialing-based lipid analyses from the same yeast cultivations using the high-throughput D48 approach, but optimisation efforts are needed.

## Supplementary Information

Below is the link to the electronic supplementary material.

Supplementary file1 (DOCX 1893 KB)

## Data Availability

All raw data associated with this manuscript are available on GNPS (10.25345/C5BN9X73B), with other associated files available on Zenodo (10.5281/zenodo.7299206) as detailed in the manuscript.

## Abbreviations

ACN: Acetonitrile
ANOVA: Analysis of variance
au: Arbitrary unit
CV: Coefficient of variance
D48: Deep-48 well method
DDA: Data-dependent acquisition
EIC: Extracted ion chromatogram
FAD: Flavin adenine dinucleotide
FC: Fold change
GC: Gas chromatography
HILIC: Hydrophilic interaction liquid chromatography
HMDB: Human Metabolome Database
*ho::kan*MX: Yeast strain with target open reading frame (HO gene) replaced by the *kan*MX resistance cassette. In this work also referred to as “wild-type”
HPLC: High-pressure liquid chromatography
HRMS: High-resolution mass spectrometry
IS: Internal standard
KEGG: Kyoto Encyclopedia of Genes and Genomes
KO: Knockout
LC: Liquid chromatography
*m/z*: Mass-to-charge ratio
MeOH: Methanol
MS^2^: Tandem mass spectrometry
MTBE: Methyl *tert*-butyl ether
NAD^+^: Nicotinamide adenine dinucleotide (oxidized form)
NEG: Negative ionization mode
Norm.: Normalized
PAVE: Peak annotation and verification engine
PCA: Principal component analysis
POS: Positive ionization mode
RP: Reverse phase
rpm: Revolutions per minute
RT: Retention time
*S. cerevisiae*: *Saccharomyces cerevisiae*, Budding yeast
SD: Standard deviation
*sdh1*Δ: Yeast strain with single gene deletion for the flavoprotein subunit of succinate dehydrogenase. In this work also referred to as “knockout”
SF: Shake flask
ESM: Electronic supplementary material
SIL: Stable isotope labelling
TOF: Time-of-flight
YMDB: Yeast Metabolome Database
YNB: Yeast nitrogen base

## Code availability

The major software used in this work, namely, PAVE2.0 (https://github.com/xxing9703/PAVE2.0), MS-DIAL (http://prime.psc.riken.jp/compms/msdial/main.html), MetFrag R Script (10.5281/zenodo.7299206, F10), and SIRIUS CSI:FingerID (https://bio.informatik.uni-jena.de/software/sirius/), are open-source. The code and files associated with this manuscript are provided in GNPS (10.25345/C5BN9X73B), and Zenodo (10.5281/zenodo.7299206).
