# Supplementary material for: High-throughput Saccharomyces cerevisiae cultivation method for credentialing-based untargeted metabolomics
Source: Anal Bioanal Chem. 2023 May 22;415(17):3415–34. doi: 10.1007/s00216-023-04724-5 (PMC10289995; doi:10.1007/s00216-023-04724-5)
Supplement: Supplementary file 1 — Supplementary file1 (DOCX 1893 KB) [file 216_2023_4724_MOESM1_ESM.docx]

# **Supplementary Information**

**High-throughput *Saccharomyces cerevisiae* cultivation method for credentialing-based untargeted metabolomics**

Lorenzo Favilli^1^*, Corey M. Griffith^1^, Emma L. Schymanski^1†^, Carole L. Linster^1†^

^1^Luxembourg Centre for System Biomedicine (LCSB), University of Luxembourg, Avenue du Swing 6, L-4367 Belvaux, Luxembourg

^†^These authors share senior authorship. *Corresponding author

[**Supplementary Information** 1](#_Toc132634323)

[**Section 1. Physiological Constrains** 2](#_Toc132634324)

[***Fig. S1:*** 2](#_Toc132634325)

[***Fig. S2:*** 3](#_Toc132634326)

[***Fig. S3****:* 3](#_Toc132634327)

[**Section 2. Credentialing-based Lipid Analysis** 4](#_Toc132634328)

[***Table S1*** 4](#_Toc132634329)

[***Table S2:*** 5](#_Toc132634330)

[**Section 2.1. Lipid Annotation** 6](#_Toc132634331)

[***Table S3*** 6](#_Toc132634332)

[***Fig. S4:*** 7](#_Toc132634333)

[**Section 3. Software Parameters** 8](#_Toc132634334)

[**Parameter Settings: MS-DIAL HILIC-BEH AMIDE, Positive and Negative Ionization Mode** 8](#_Toc132634335)

[**Parameter Settings: MS-DIAL CSH18-Lipids Analysis, Positive and Negative Ionization Mode** 15](#_Toc132634336)

[**PAVE Parameters** 23](#_Toc132634337)

[**Proteowizard MSConvert** 25](#_Toc132634338)

[**mzXML Data Conversion** 25](#_Toc132634339)

[**mzML Conversion** 26](#_Toc132634340)

[**Shinyscreen** 26](#_Toc132634341)

[**SIRIUS CSI:FingerID** 27](#_Toc132634342)

[**Thermo Scientific Xcalibur: Manual Mass Shift Quality Control** 28](#_Toc132634343)

[**References** 29](#_Toc132634344)

# **Section 1. Physiological Constraints**

**Fig. S1:** Growth of yeast WT strain in the SF (black) and D48 (red) experimental setups. Glucose consumption of WT yeast in SF (blue) and D48 (green) cultivations. The violet (dashed) line shows the sampling point for the LC-MS based metabolomics analysis (conducted in an independent experiment). Each point of the curve refers to means ±SDs for three biological replicates.

The linearity range of the growth and glucose consumption curve was used to calculate the growth rate *μ* (1/h) and the glucose uptake rate (-mM/h) for the yeast WT in both experimental setups.

**Fig. S2:** Linearity range of the growth curve of WT yeast for the SF (black) and D48 (red) cultivation. The growth rate is 0.37 h^-1^ in the D48 and 0.36 h^-1^ in the SF experimental setup. Each point of the curve refers to means ±SDs for three biological replicates.

**Fig. S3**: Linearity range of the glucose consumption of WT yeast for the SF (blue) and D48 (green) cultivation. The rate of the glucose consumption is 4.17 mM∙ h^-1^ in the D48 and 3.45 mM∙ h^-1^ in the SF experimental setup. Each point of the curve refers to means ±SDs for three biological replicates.

# **Section 2. Credentialing-based Lipid Analysis**

The credentialing data analysis for the lipids data using the PAVE workflow [3] with the adapted parameters was performed separately for each strain and experimental condition tested (SF-WT, SF-KO, D48-WT, and D48-KO).

**Table S1:** Feature statistics following PAVE supported analysis of the RP-HRMS lipid data acquired in positive ionization mode.

| **PAVE Output** | **RP-Lipids, Positive Ionization Mode** | | | |
| --- | --- | --- | --- | --- |
|  | **D48-WT** | **D48-KO** | **SF-WT** | **SF-KO** |
| Total Detected Features | 18290 | | | |
| Adduct | 383 | 363 | 358 | 371 |
| Background | 15894 | 15596 | 16581 | 16433 |
| Dimer | 2 | 6 | 4 | 1 |
| Fragment | 3 | 3 | 7 | 7 |
| Heterodimer | 0 | 6 | 3 | 0 |
| Isotope | 108 | 153 | 94 | 94 |
| Low Carbon Count | 117 | 155 | 58 | 62 |
| Low Score | 921 | 1088 | 383 | 461 |
| Multicharge | 13 | 11 | 6 | 6 |
| Discarded Features | 17441 | 17381 | 17494 | 17435 |
| Discarded Features (%) | 95.4 % | 95 % | 96 % | 95.3 |
| Credentialed Features | 849 | 909 | 769 | 855 |
| Credentialed Features (%) | 4.6 % | 5 % | 4 % | 4.7 % |
| Total Unique Credentialed Features per Setup | 1361 | | 1087 | |
| Total Unique Credentialed Features per Setup (% vs. Total Detected Features) | 7.4 % | | 6 % | |
| ***Retained Features**** | **289** | | **164** | |
| ***Retained Features (%)*** | **1.6 %** | | **1.5 %** | |

* Credentialed features that passed mass-shift quality control criteria in Shinyscreen (*i.e.*, showing a corresponding mass shift in the uniformly labeled ^13^C^15^N cultivation conditions)

**Table S2:** Feature statistics following PAVE supported analysis of the RP-HRMS lipid data acquired in negative ionization mode.

| **PAVE Output** | **RP-Lipids, Negative Ionization Mode** | | | |
| --- | --- | --- | --- | --- |
|  | **D48-WT** | **D48-KO** | **SF-WT** | **SF-KO** |
| Total Detected Features | 9265 | | | |
| Adduct | 56 | 55 | 38 | 38 |
| Background | 8571 | 8555 | 8684 | 8622 |
| Dimer | 4 | 4 | 0 | 0 |
| Fragment | 18 | 13 | 42 | 42 |
| Heterodimer | 2 | 1 | 1 | 0 |
| Isotope | 76 | 78 | 85 | 81 |
| Low Carbon Count | 25 | 47 | 8 | 9 |
| Low Score | 49 | 59 | 35 | 59 |
| Multicharge | 0 | 0 | 0 | 0 |
| Discarded Features | 8801 | 8812 | 8893 | 8851 |
| Discarded Features (%) | 95 % | 95.1 % | 96 % | 95.5 % |
| Credentialed Features | 464 | 453 | 372 | 414 |
| Credentialed Features (%) | 5 % | 4.9 % | 4 % | 4.5 % |
| Total Unique Credentialed Features per Setup | 604 | | 520 | |
| Total Unique Credentialed Features per Setup  (% vs. Total Detected Features) | 6.5 % | | 5.6 % | |
| ***Retained Features**** | **109** | | **68** | |
| ***Retained Features (%)*** | **1.2 %** | | **0.7 %** | |

* Credentialed features that passed mass-shift quality control criteria in Shinyscreen (*i.e.*, showing a corresponding mass shift in the uniformly labeled ^13^C^15^N cultivation conditions)

## **Section 2.1. Lipid Annotation**

The credentialed features that passed the mass-shift criteria in Shinyscreen of the lipid analysis (SI, Section 2, Table S.1-2; 1.6% and 1.5% credentialed features in D48 and SF, respectively, out of the 18290 total features detected in positive ionization mode; 1.2% and 0.7% credentialed features in D48 and SF, respectively, out of the 9265 total features detected in negative ionization mode) were annotated only using MS-DIAL and reported as level 3 by *in silico* database matching, and the quality of the credentialing/annotation analysis results was verified manually. Features for which a mass shift could not be confirmed upon this manual inspection were not annotated and reported as false positive credentialed features (% vs. total amount of credentialed features, Table S3).

**Table S3:** Annotation results for the credentialed features of the lipid analysis. False Positive percentage refer to the ratio of the annotated features in which a mass shift could not be confirmed manually in the labeled .raw data to the total amount of annotated features prior the mass shift quality control

| **Lipid Analysis, Positive Ionization** | | **Confidence Level** | **Lipid Analysis, Negative Ionization** | |
| --- | --- | --- | --- | --- |
| **D48** | **SF** |  | **D48** | **SF** |
| 34 | 32 | Level 3 MS-DIAL | 26 | 35 |
| 113 | 70 | Level 5 | 20 | 31 |
| 147 | 102 | Total | 46 | 66 |
| 10.9 | 9.7 | **% False Positive**  **(No-Mass Shift/Total Annotated)** | 4.2 | 4.3 |

One example of credentialed lipid molecule, putatively annotated as 1-tetradecyl-2-acetyl-sn-glycero-3-phosphocholine (PC (O-16:0), is shown in Figure S4 with related mass shift across the labeled conditions and match between the experimental and predicted MS^2^ spectra.


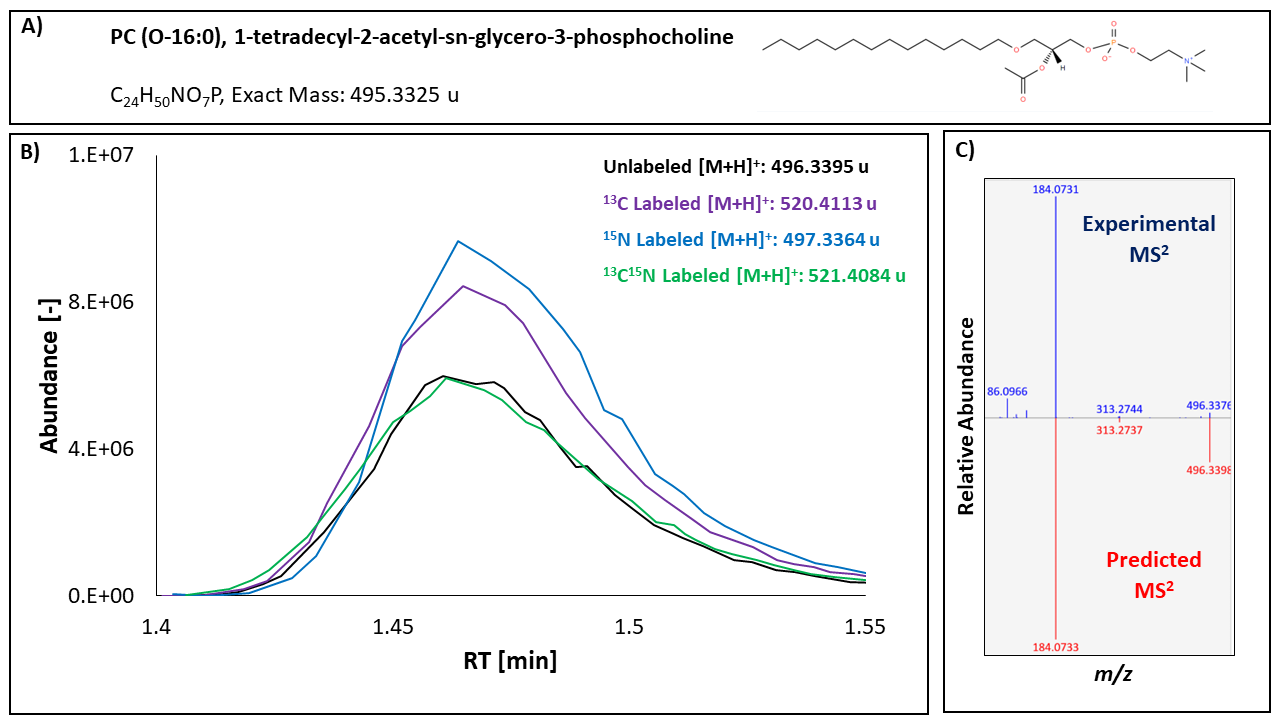


**Fig. S4:** Extracted Ion Chromatogram (EIC) of an annotated peak from (un)labeled nonpolar cell extracts and related predicted MS^2^ spectral match**.** (A) 1-tetradecyl-2-acetyl-sn-glycero-3-phosphocholine (PC (O-16:0), chemical formula, exact mass, and chemical structure. (B) EICs for the putatively annotated PC (O-16:0) in unlabeled (black), ^13^C-(purple), ^15^N-(light blue) and ^13^C^15^N-cell extracts (green) in the positive ionisation mode. (C) Match between the measured MS^2^ spectrum (blue) and the in silico predicted MS^2^spectrum (red).

# **Section 3. Software Parameters**

## **Parameter Settings: MS-DIAL HILIC-BEH AMIDE, Positive and Negative Ionization Mode**


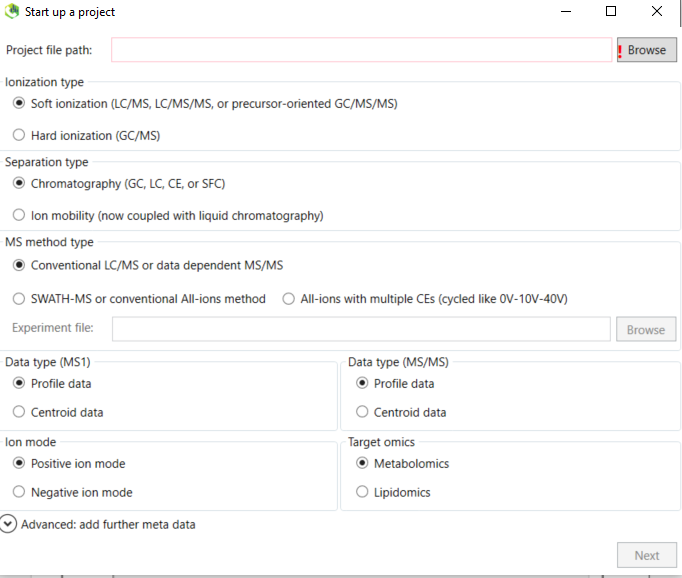


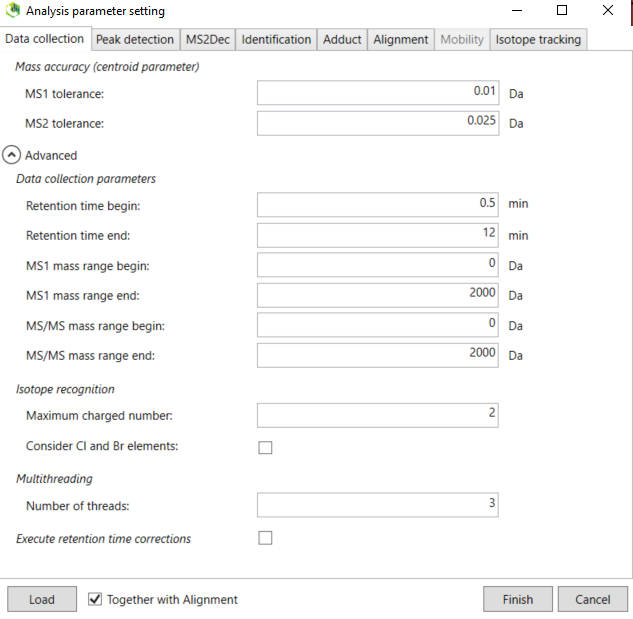


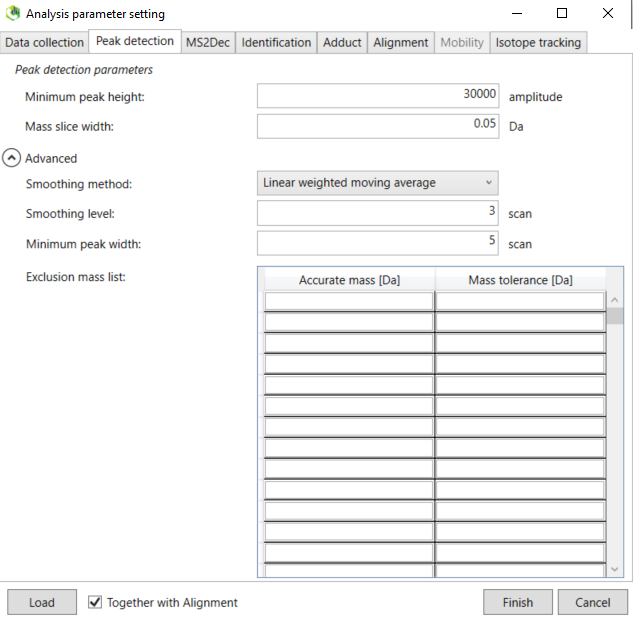


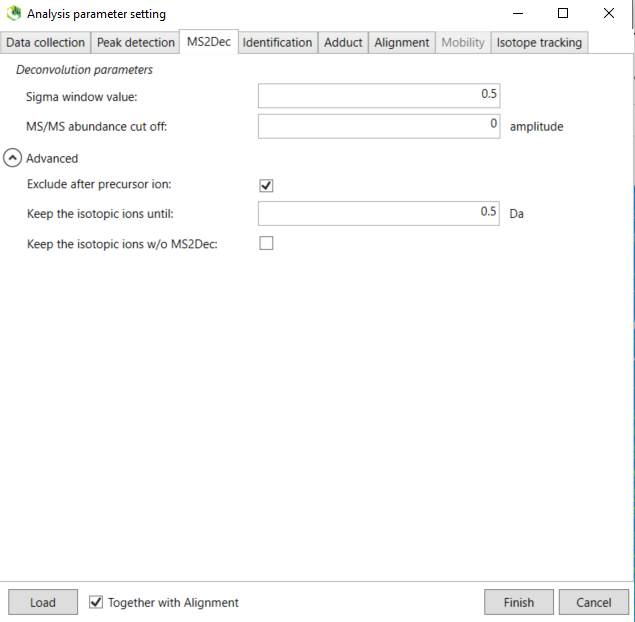


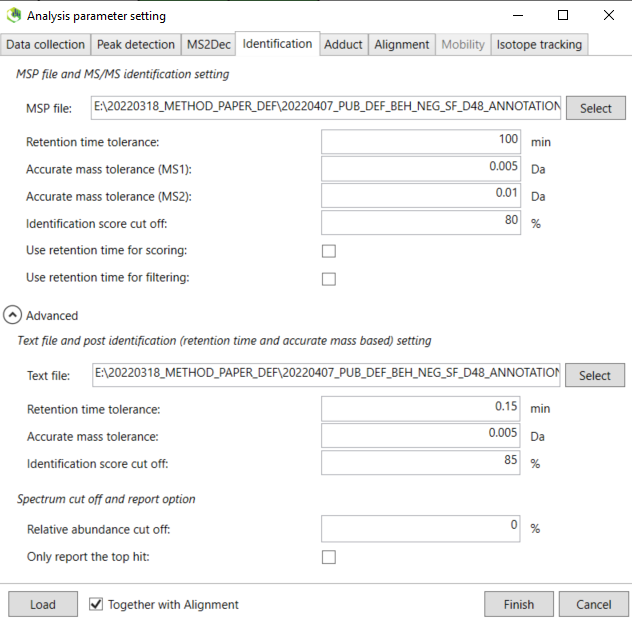


*The Identification score cut-off was bypassed manually in the subsequent annotation step using MS-DIAL and applied only to credentialed features that passed the Shinyscreen quality control procedure. For the annotation of HILIC data with MS-DIAL a feature was annotated as “Level 2A MS-DIAL” manually if it fulfilled the minimal criteria of a dot product ≥ 50 % and fragment presence ≥ 50 % (see Materials and Methods, Data Analysis section in the main text).


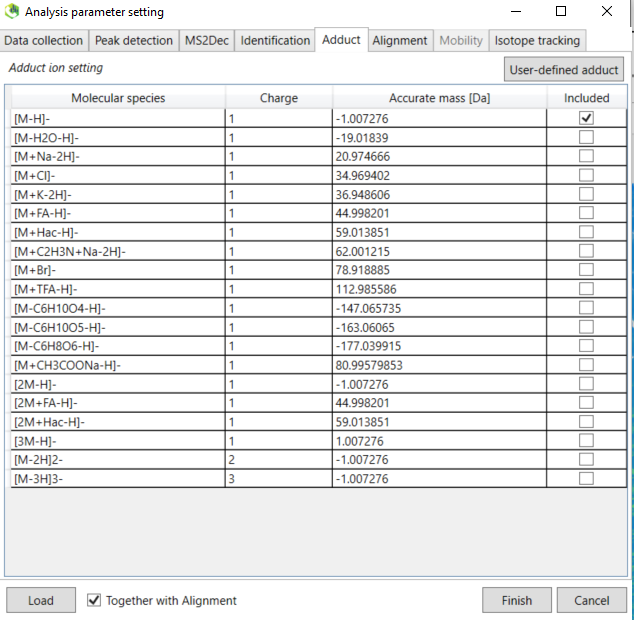


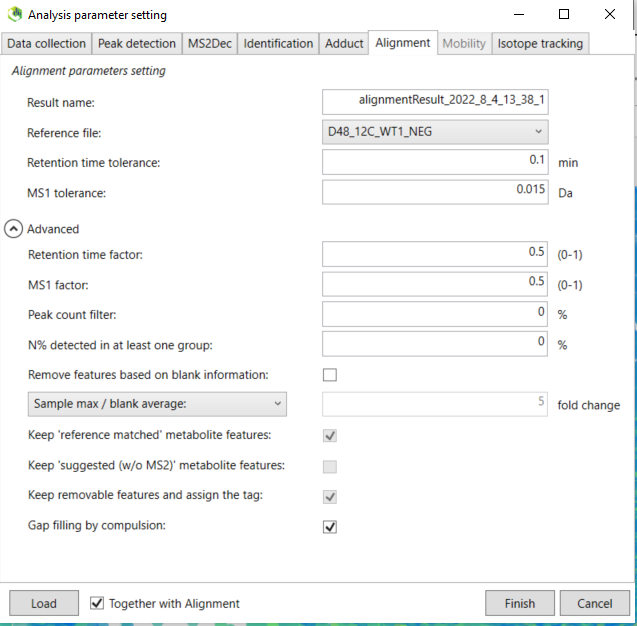


## **Parameter Settings: MS-DIAL CSH18-Lipids Analysis, Positive and Negative Ionization Mode**


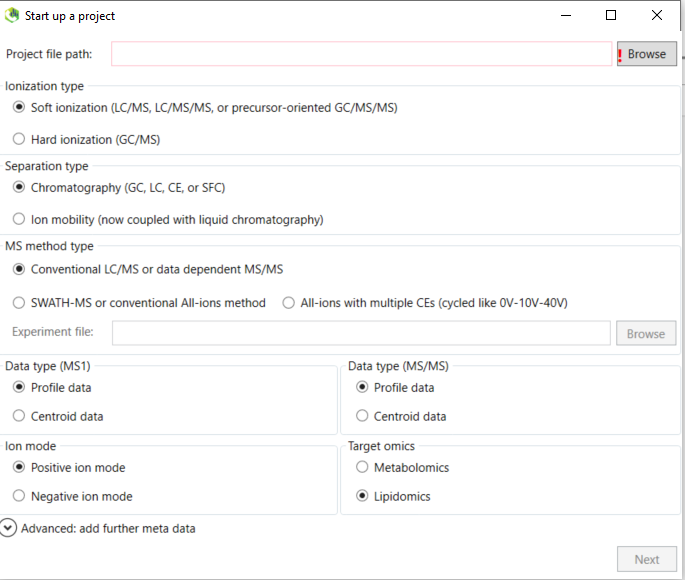


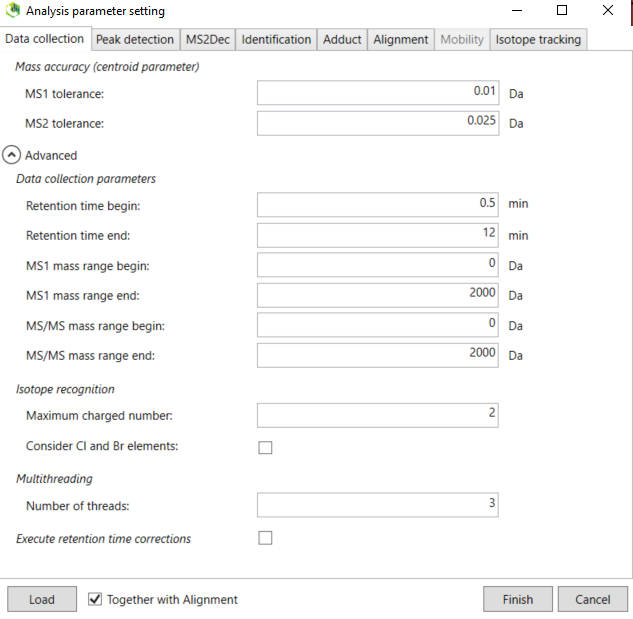


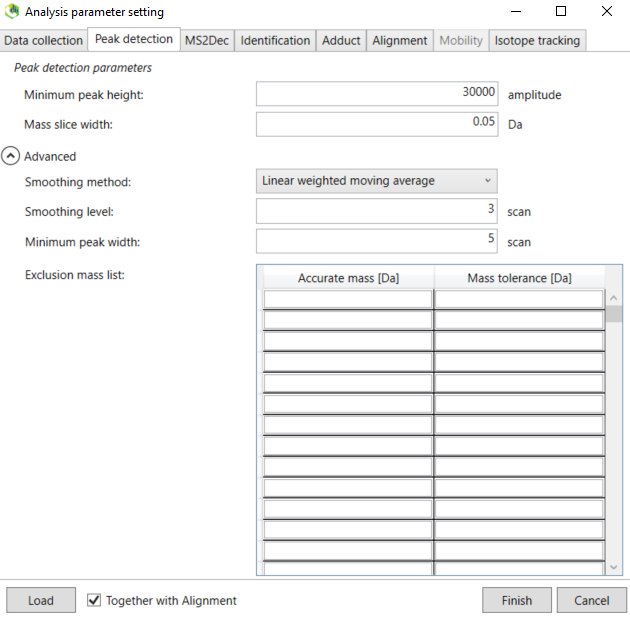


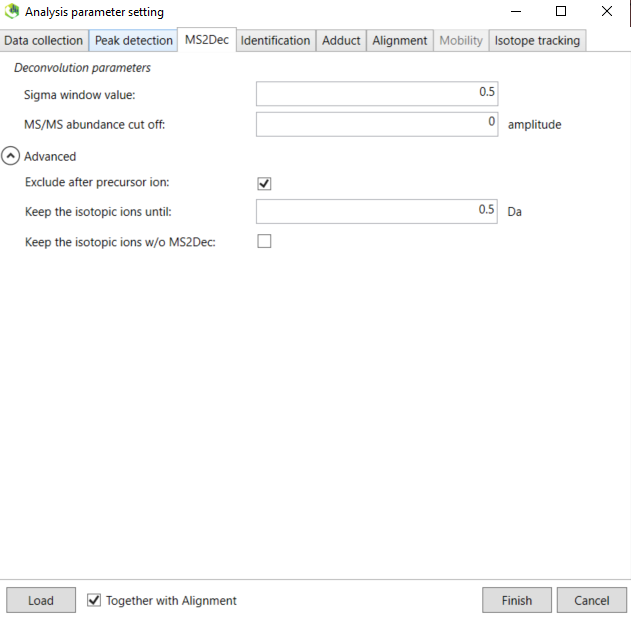


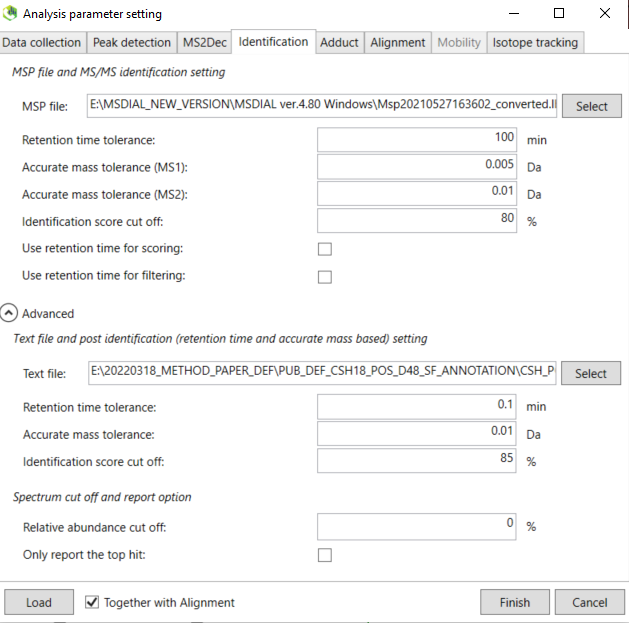


*The Identification score cut-off was bypassed manually in the subsequent annotation step using MS-DIAL and applied only to credentialed features that passed the Shinyscreen quality control procedure. For the annotation of lipids data with MS-DIAL a feature was annotated as “Level 3 MS-DIAL” manually if it fulfilled the minimal criteria of a dot product ≥ 40 % (see Materials and Methods, Data Analysis section in the main text).


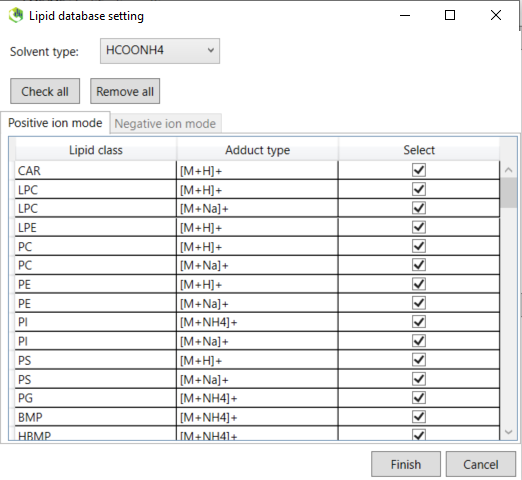


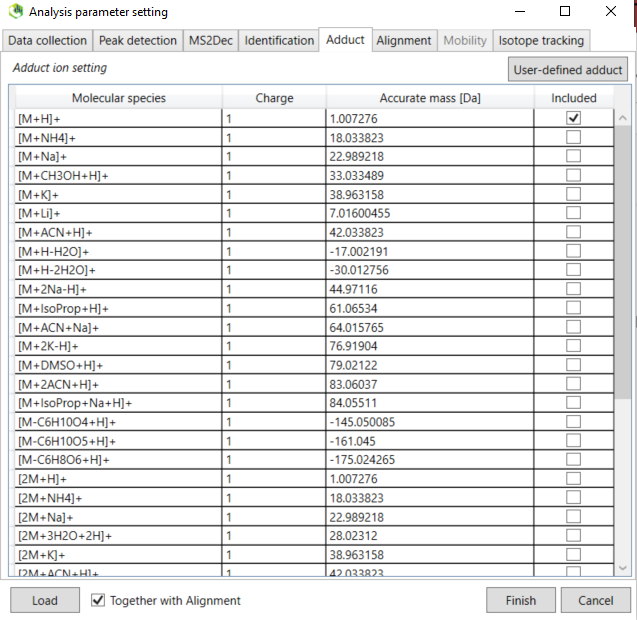


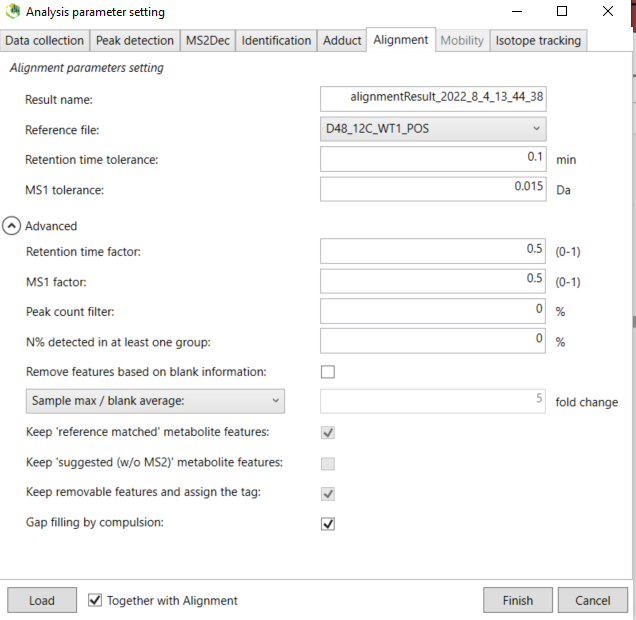


## **PAVE Parameters**


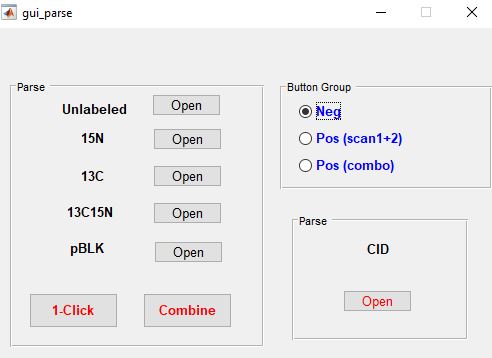


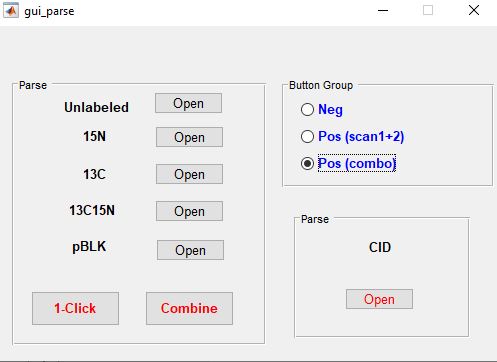


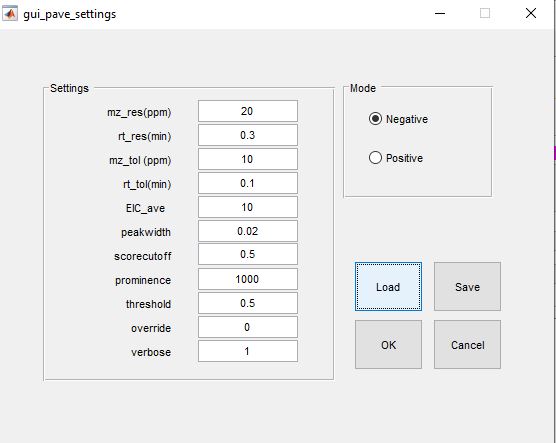


## **Proteowizard MSConvert**

### **mzXML Data Conversion**


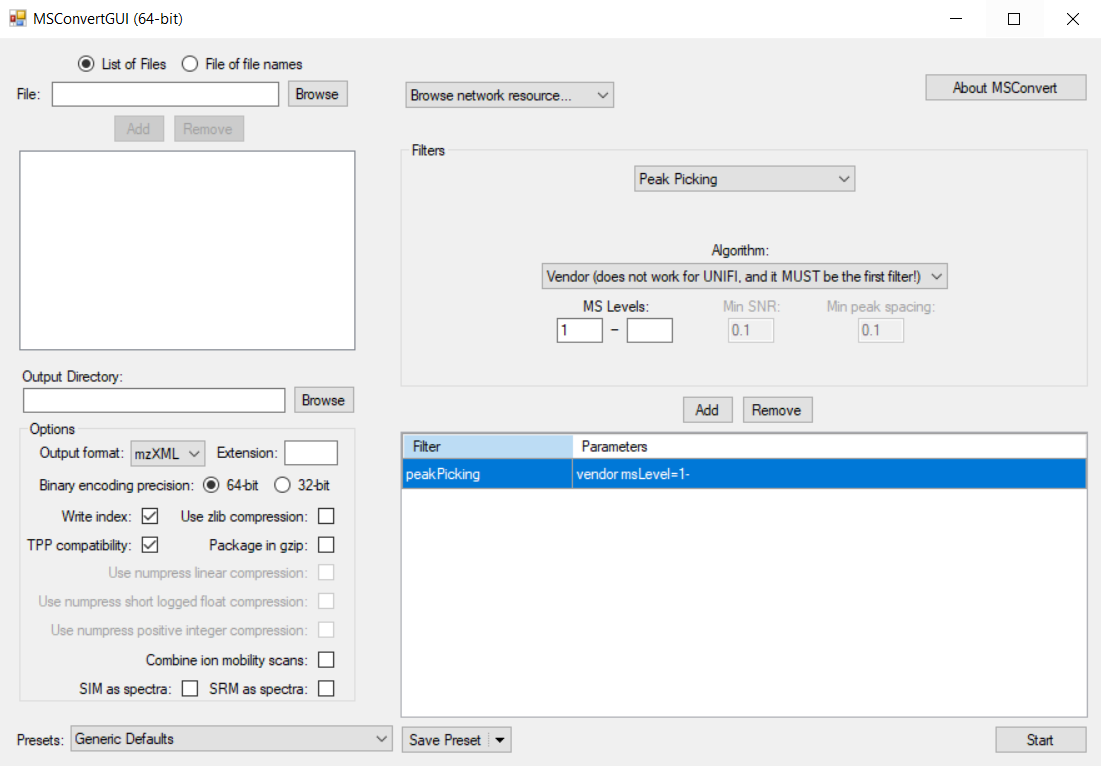


### **mzML Conversion**


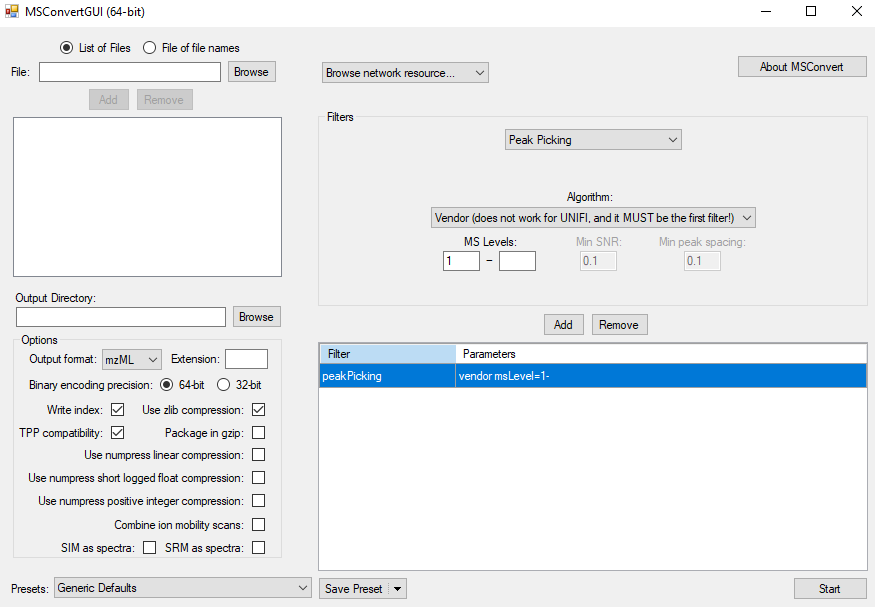


## **Shinyscreen**


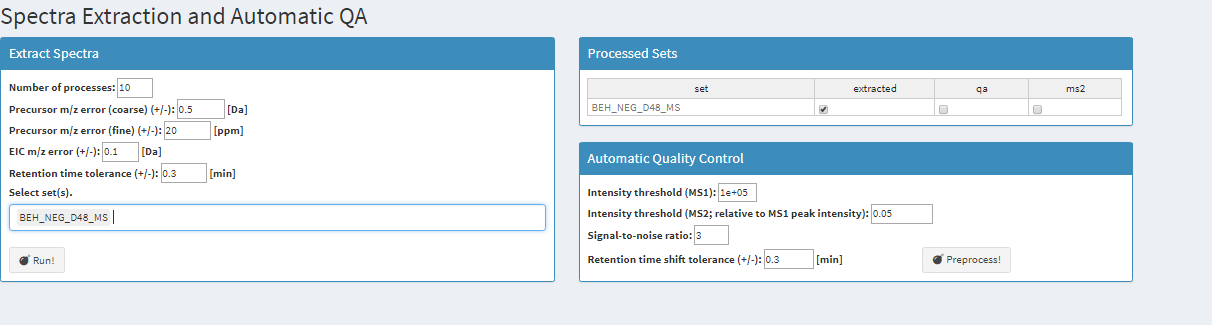


## **SIRIUS CSI:FingerID**


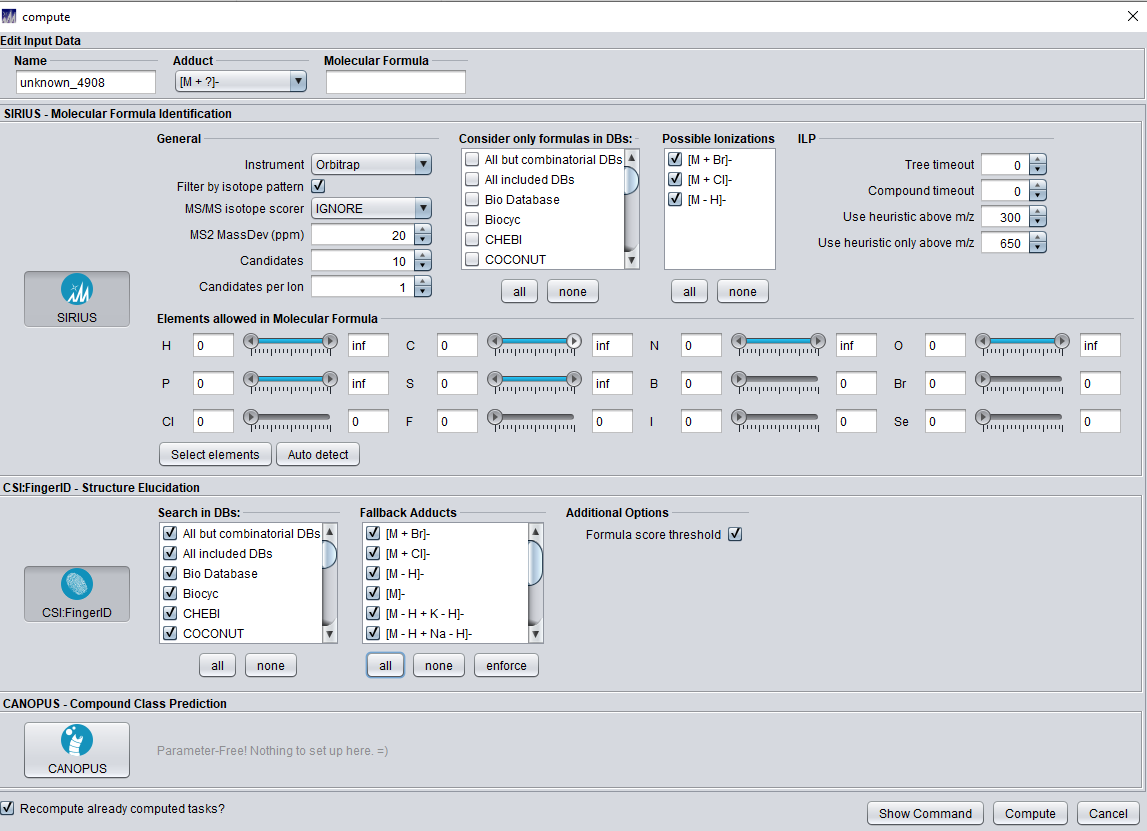


## **Thermo Scientific Xcalibur: Manual Mass Shift Quality Control**


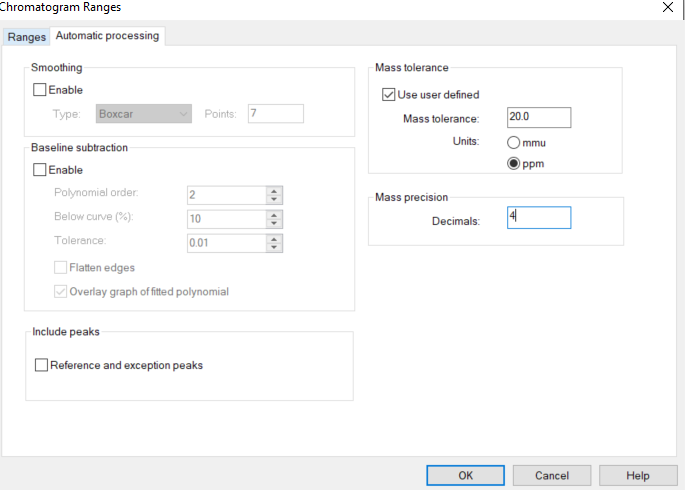


# **References**

1. Gibney PA, Lu C, Caudy AA, Hess DC, Botstein D (2013) Yeast metabolic and signaling genes are required for heat-shock survival and have little overlap with the heat-induced genes. Proc Natl Acad Sci 110:. https://doi.org/10.1073/pnas.1318100110

2. Ewald JC, Heux S, Zamboni N (2009) High-Throughput Quantitative Metabolomics: Workflow for Cultivation, Quenching, and Analysis of Yeast in a Multiwell Format. Anal Chem 81:3623–3629. https://doi.org/10.1021/ac900002u

3. Wang L, Xing X, Chen L, Yang L, Su X, Rabitz H, Lu W, Rabinowitz JD (2019) Peak Annotation and Verification Engine for Untargeted LC–MS Metabolomics. Anal Chem 91:1838–1846. https://doi.org/10.1021/acs.analchem.8b03132

4. Blaženović I, Kind T, Sa MR, Ji J, Vaniya A, Wancewicz B, Roberts BS, Torbašinović H, Lee T, Mehta SS, Showalter MR, Song H, Kwok J, Jahn D, Kim J, Fiehn O (2019) Structure Annotation of All Mass Spectra in Untargeted Metabolomics. Anal Chem 91:2155–2162. https://doi.org/10.1021/acs.analchem.8b04698
